# Ataluren‐Induced Functional Restoration of Neurofibromin in Fibroblasts From Neurofibromatosis Type 1 Patients With Nonsense Mutations

**DOI:** 10.1002/mco2.70485

**Published:** 2025-11-16

**Authors:** Soyoung Kim, Hyosang Do, Sun Hee Heo, Minji Kang, Soojin Hwang, Dohyung Kim, Min‐Hoo Chang, Kyung Kim, Beom Hee Lee

**Affiliations:** ^1^ Asan Institute for Life Sciences Asan Medical Center University of Ulsan College of Medicine Seoul Republic of Korea; ^2^ Department of Pediatrics Asan Medical Center Children's Hospital University of Ulsan College of Medicine Seoul Republic of Korea; ^3^ Medical Genetics Center Asan Medical Center University of Ulsan College of Medicine Seoul Republic of Korea; ^4^ Humanscape Inc Seoul Republic of Korea; ^5^ Genoscape Pte. Ltd. South Beach Tower Singapore

**Keywords:** AMPD3, ataluren, neurofibromatosis type 1 (NF1), nonsense mutations, restored neurofibromin, TGFBR3

## Abstract

Neurofibromatosis Type 1 (NF1) is an autosomal dominant genetic disorder caused by heterogeneous mutations in the tumor suppressor gene *NF1*. Neurofibromin, encoded by *NF1*, predominantly acts as a negative regulator of the RAS‐MEK signaling pathway. Up to 30% of NF1 patients harbor nonsense mutations (NS) that introduce premature termination codons (PTCs). Ataluren is a well‐characterized small molecule that acts as a nonsense suppressor by enhancing the ribosomal readthrough of PTCs. Here, we isolated primary fibroblasts from 22 Korean *NF1^NS/+^* patients and comprehensively evaluated the efficacy of ataluren treatment. The results demonstrate that hyperactivated GTP‐bound RAS was significantly alleviated in approximately 23% of *NF1^NS/+^* fibroblasts, and the cellular levels of phosphorylated ERK also decreased in approximately 24% after ataluren treatment. Through transcriptome‐wide profiling based on ataluren responsiveness, we analyzed a subset of genes in ataluren‐treated *NF1^NS/+^* fibroblasts whose expression was significantly altered in ataluren‐responsive cells, but not in nonresponsive cells. Furthermore, both AMPD3 and TGFBR3 were notably identified as feasible biomarkers for monitoring functional neurofibromin. Interestingly, AMPD3 can be an effective therapeutic target for NF1‐associated diseases. Together, our study suggests that ataluren can be considered a therapeutic agent for some *NF1^NS/+^* patients and contributes to expanding insights into NF1 therapy.

## Graphical abstract

The therapeutic potential of ataluren for NF1 nonsense mutations was assessed by a comprehensive evaluation of the RAS/MEK/ERK pathway activity in 22 *NF1^NS/+^* patient‐derived fibroblasts. The whole‐transcriptomic profiles between ataluren responders and nonresponders, and further analysis and validation revealed novel biomarkers, AMPD3 and TGFBR3, for monitoring NF1 therapy and highlighted AMPD3 as a new therapeutic target for NF1‐associated diseases.

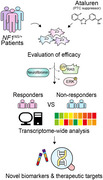

## 1 Introduction

Neurofibromatosis type 1 (NF1) is one of the most common genetic diseases, affecting 1 in 3000 births, caused by heterogeneous mutations in the tumor suppressor gene *NF1* [1]. Neurofibromin, encoded by *NF1*, regulates cell growth and survival by inhibiting the accumulation of hyperactive RAS (GTP‐bound RAS), while dysfunctional neurofibromin induces cell growth‐promoting signals such as the MEK‐ERK effector pathway [2, 3]. Because multiple organ systems, such as skin, brain, and sciatic nerve, are affected in NF1, affected patients exhibit variable clinical manifestations, including pigmentary lesions, skeletal abnormalities, peripheral or central nervous system tumors, and learning difficulties [3, 4]. Neurofibroma is a benign nerve sheath tumor that affects almost all NF1 patients. Importantly, up to 50% of NF1 patients develop plexiform neurofibroma (PN), of which 8%–13% are at high risk for malignant transformation [4]. While the standard treatment for PN used to be surgical resection, the MEK inhibitor selumetinib has recently gained attention by showing a 20% or higher volume reduction of inoperable PN in over 70% of treated patients [5, 6, 7]. Despite selumetinib treatment, about 30% of patients with inoperable PN do not respond to selumetinib, necessitating further investigation for alternative therapeutic drugs for NF1 patients.

Nonsense mutations are in‐frame point mutations that introduce premature termination codons (PTCs) and are responsible for approximately 10% of genetic disorders [8]. Notably, approximately 20%–30% of NF1 patients have nonsense mutations in the *NF1* gene [9, 10], indicating that effective PTC suppressors (e.g., ataluren) could be considered as potential therapeutic agents for those patients.

Ataluren (also known as PTC124) is a small molecule that selectively induces ribosomal readthrough of PTCs, but not that of normal termination codons [11]. Importantly, ataluren exhibits minimal off‐target effects, as evidenced by its selective action on target proteins without inducing aberrant readthrough products, which allows for long‐term administration [11]. Consequently, its potential usage has been considered for the treatment of Duchenne muscular dystrophy [12]. Recent studies have also assessed the therapeutic potential of ataluren in mouse and minipig models of *NF1*‐carrying PTCs [10, 13, 14]. These reports demonstrated that PTC suppressors, such as ataluren, G418, and gentamicin, can improve functional neurofibromin, which results in the reduction of phosphorylated ERK (p‐ERK) levels in mouse differentiated neuronal cells [13], in primary Schwann cells isolated from minipig neurofibromas [10], and moderately alleviating neurofibroma growth and paralysis phenotypes in a mouse model with nonsense mutation [14].

In the current study, fibroblasts were isolated from 22 Korean NF1 patients harboring different nonsense mutations (*NF1^NS/+^*), in which the readthrough efficiency of ataluren was determined by measuring GTP‐bound RAS activities and cellular p‐ERK levels. This study predicted different responses among the *NF1^NS/+^* fibroblasts. The whole‐transcriptome profiles were also distinctive between ataluren‐responsive and nonresponsive *NF1^NS/+^* fibroblasts. Further analysis and validation revealed new biomarkers for monitoring NF1 therapy and identified novel therapeutic targets.

## 2 Results

### 2.1 Patients With Nonsense Mutations in *NF1*

**FIGURE 1:**
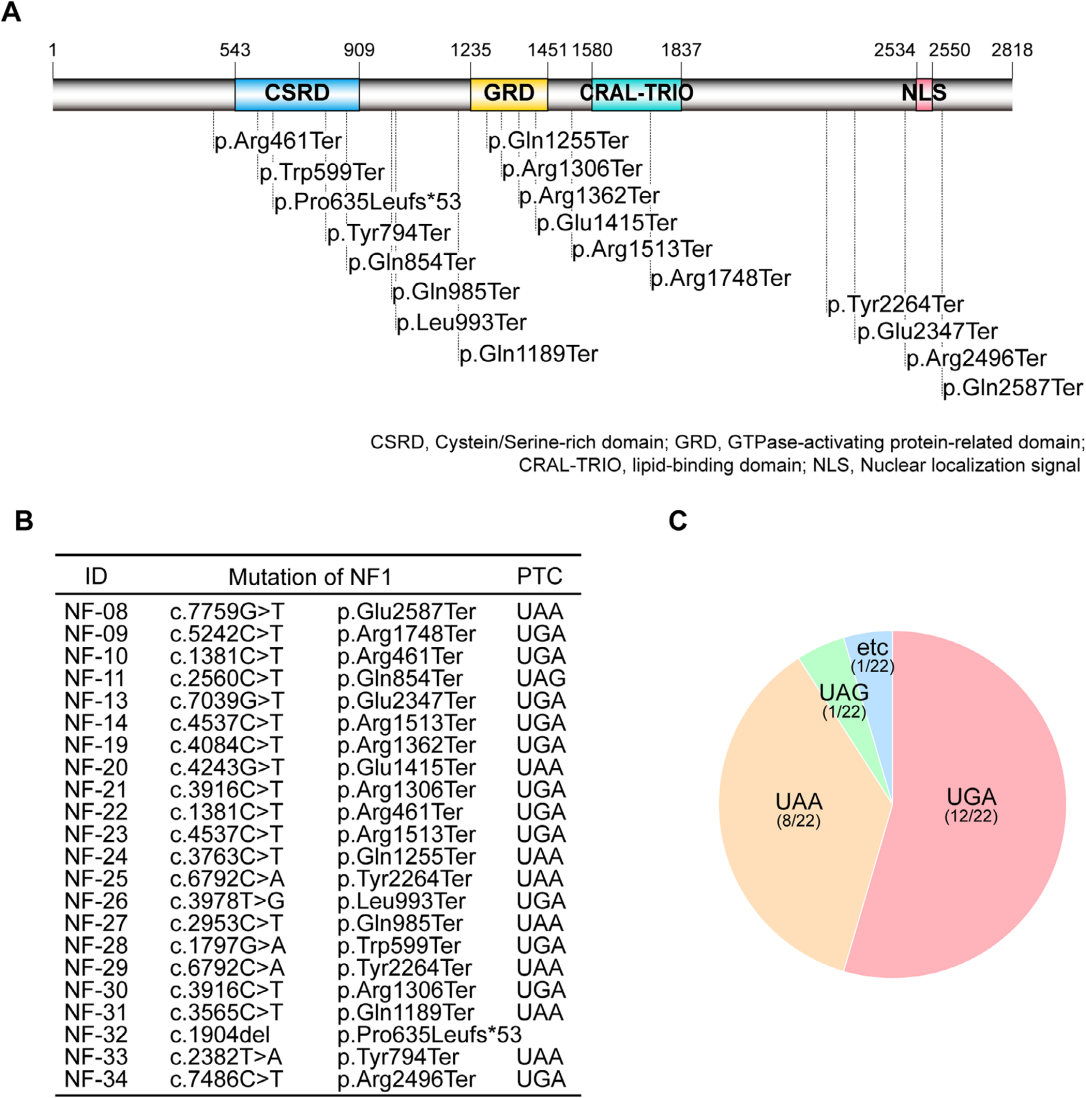
Genetic information of 22 Korean *NF1^NS/+^* patients with nonsense mutations. (A) Mutated sites of 22 *NF1^NS/+^* patients are indicated on the domain architecture of neurofibromin. (B) Mutations (*n* = 18) were identified in 22 *NF1^NS/+^* patients harboring PTCs on the *NF1* gene. (C) Distribution of PTC types (UGA, UAA, UAG) among 22 *NF1^NS/+^* patients.

In our previous study, 28.9% of Korean NF1 patients had nonsense mutations in *NF1* that led to PTCs [9]. The clinical profiles and characteristics of 22 Korean *NF1^NS/+^* patients included in the study are summarized in Tables S1 and S2. Specifically, the mutation sites of 22 *NF1^NS/+^* patients were evenly distributed along the *NF1* coding sequences, resulting in the truncated form of neurofibromin (Figure 1A, B). Notably, 18 different mutation spots were identified, including four recurrent sites (p.Arg461Ter, p.Arg1306Ter, p.Arg1513Ter, and p.Tyr2264Ter). Since ataluren, a small molecule that suppresses PTCs, has the highest readthrough at UGA [11], we investigated the distribution of generated PTC in the patients. The data showed that a UGA termination codon accounted for approximately 54.5%, followed by UAA (36.4%) and UAG (4.5%) (Figure 1C). Therefore, we hypothesized that ataluren treatment could feasibly restore the full‐length neurofibromin in patients harboring nonsense mutations in *NF1*.

### 2.2 Evaluation of the Efficacy of Ataluren in *NF1^NS/+^* Fibroblasts

**FIGURE 2:**
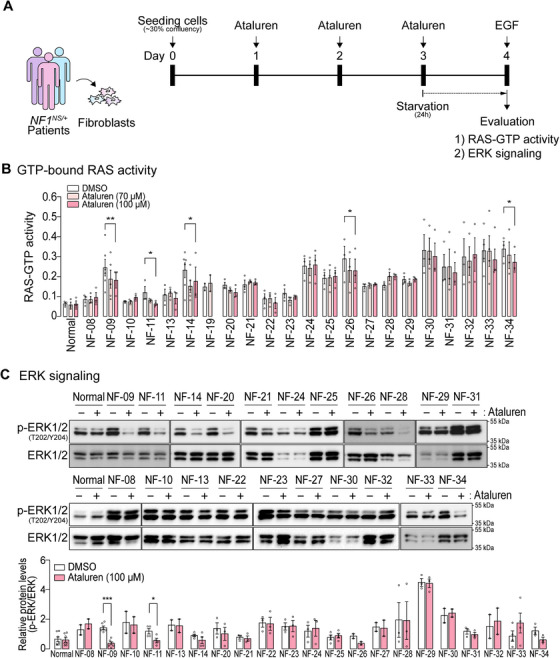
Hyperactive RAS (GTP‐bound RAS) signaling and ERK activities are decreased in *NF1^NS/+^* patient fibroblasts after ataluren treatment. (A) Experimental strategy for evaluating ataluren efficacy using *NF1^NS/+^* patient fibroblasts. The fibroblasts were treated with or without ataluren (70 or 100 µM) for 72 h. (B) GTP‐bound RAS activities of fibroblasts treated with ataluren or DMSO control were measured using a G‐LISA Ras activation assay. The graphs are displayed as mean ± SEM. Student's paired *t*‐test with Wilcoxon rank tests were performed between DMSO control and ataluren (100 µM)‐treated samples (*p < 0.05, **p < 0.01). (C) ERK activities assessed by measuring the levels of p‐ERK normalized to those of total ERK in Western blot analysis. The protein abundance of the p‐ERK/ERK ratio was quantified and indicated in the graph below. Two‐way ANOVA with Sidak's multiple comparisons test was conducted (*p < 0.05, ***p < 0.001).

Fibroblasts from 22 *NF1^NS/^* ^+^ patients and a normal control were isolated from the skin. The isolated primary fibroblasts exhibited a strong expression of vimentin, a representative fibroblast marker, while the expression of E‐cadherin, an epithelial marker, was markedly lower compared to that in the epithelial HEK293 cells (Figure S1). Both GTP‐bound RAS and ERK activities were measured in the ataluren‐treated fibroblasts (Figure 2A). In the absence of ataluren, the basal levels of active RAS (RAS‐bound GTP) were elevated in *NF1^NS/+^* fibroblasts compared with normal controls, irrespective of the location of the mutation sites on *NF1* (Figure 2B). Following ataluren treatment, GTP‐bound RAS activities were significantly reduced in 22.7% of the total 22 *NF1^NS/+^* patient fibroblasts, including five fibroblasts harboring a UGA or UAG premature stop codon (NF‐09, NF‐11, NF‐14, NF‐26, and NF‐34), supporting that ataluren can directly promote the suppressing PTCs of *NF1* in vitro. Several UAA‐bearing fibroblasts (NF‐20, NF‐31, and NF‐33) showed a tendency toward decreased RAS‐GTP activities after ataluren treatment, but this effect was not as pronounced as in UGA or UAG *NF1^NS/+^* fibroblasts.

To determine whether ataluren‐induced neurofibromin had physiologically relevant functional activity in fibroblasts, we monitored the MEK‐ERK signaling cascade by assessing the ratio of p‐ERK to total ERK in the presence or absence of ataluren in 21 *NF1^NS/+^* fibroblasts. In addition to the five *NF1^NS/+^* fibroblasts in which ataluren significantly decreased active RAS levels (Figure 2B), two additional *NF1^NS/+^* fibroblasts (NF‐20 and NF‐31) demonstrated a decreasing trend in the p‐ERK/ERK ratio, despite no significant reduction in active RAS levels being observed (Figure 2C). Taken together, ataluren restored MEK‐ERK activity in 23.8% of *NF1^NS/+^* fibroblasts.

### 2.3 Transcriptome‐Wide Analysis of Ataluren Responders and Nonresponders

Since ataluren is known to have no significant effect on mRNA expression and stability [11], we examined whether ataluren altered NF1 expression through the nonsense‐mediated mRNA decay (NMD) pathway in *NF1^NS/+^* fibroblasts. Six *NF1^NS/+^* patient fibroblasts, each harboring a different location of PTC, were selected to investigate the role of NMD in regulating PTC‐bearing NF1 transcripts: NF‐09 (c.5242C>T; p.Arg1748Ter), NF‐11 (c.2560C>T; p.Gln854Ter), NF‐22 (c.1381C>T; p.Arg461Ter), NF‐23 (c.4537C>T; p.Arg1513Ter), NF‐25 (c.6792C>A; p.Tyr2264Ter), and NF‐31 (c.3565C>T; p.Gln1189Ter). Compared with normal controls, NF1 mRNA was remarkably downregulated in these *NF1^NS/+^* fibroblasts (Figure S2A). When UPF1, a key mediator of the NMD pathway [15], was depleted in these six *NF1^NS/+^* fibroblasts, NF1 mRNA levels were not significantly upregulated relative to control siRNA‐treated cells (Figure S2B). Furthermore, we observed that the expression levels of neurofibromin and p‐ERK were not considerably altered by NMD suppression between the DMSO control and ataluren‐treated fibroblasts (Figure S2C), indicating that NMD suppression did not improve the functional restoration of neurofibromin following ataluren treatment in both ataluren‐responsive (NF‐09 and NF‐11) and nonresponsive (NF‐22, NF‐23, NF‐25, and NF‐31) *NF1^NS/+^* fibroblasts. Taken together, these results demonstrate that several PTC‐bearing NF1 transcripts are not the primary targets of the NMD machinery.

**FIGURE 3:**
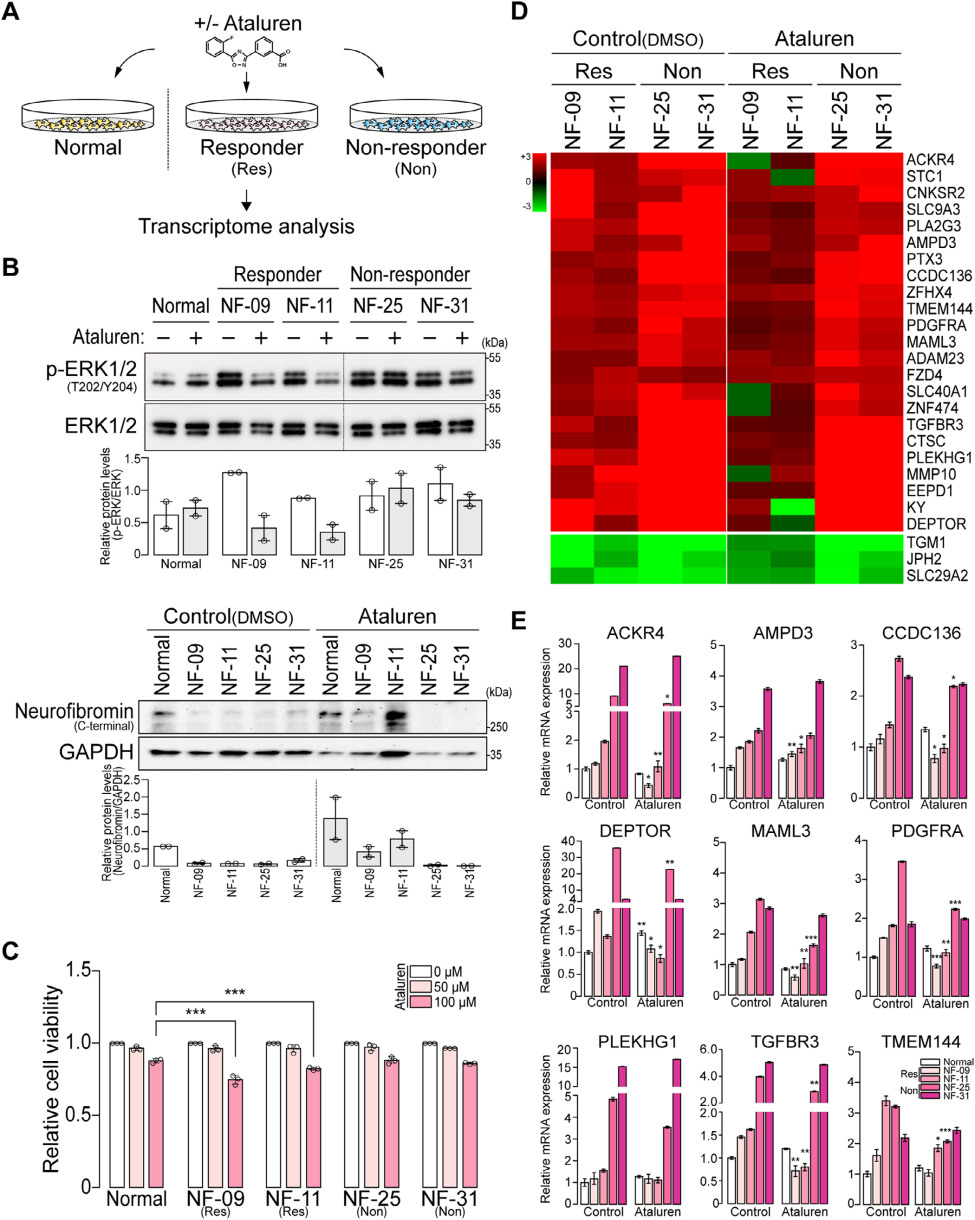
Transcriptome‐wide profiling between ataluren‐responsive and nonresponsive fibroblasts. (A) Schematic illustration of the transcriptome analysis for *NF1^NS/+^* patient fibroblasts, including ataluren responders (Res) and nonresponders (Non), and normal fibroblasts. (B) ERK signaling activity and neurofibromin levels were validated by Western blotting in two representative *NF1^NS/+^* fibroblasts for ataluren responders (NF‐09 and NF‐11) or nonresponders (NF‐25 and NF‐31). The protein levels of total ERK and GAPDH served as loading controls. The band intensities were quantified and indicated in graphs (*n *= 2; white: DMSO control, gray: 100 µM ataluren). (C) Cell viability was estimated in *NF1^NS/+^* fibroblasts after ataluren treatment (0, 50, and 100 µM). *p*‐values were calculated using two‐way ANOVA with Dunnett's multiple comparisons test (*n *= 3; normal vs. *NF1^NS/+^*). (D) Clustering heatmap analysis demonstrates the subsets of differentially expressed genes (DEGs) in ataluren‐responsive and nonresponsive fibroblasts before or after ataluren (100 µM) treatment. The color scale from red to green represents the values of log_2_ (fold change) from large to small. (E) Relative mRNA expression determined in fibroblasts treated with DMSO control or ataluren (100 µM) by quantitative PCR. The mRNA level of GAPDH was used as an internal control. The graphs are displayed as mean ± SEM (*n *= 3). *p*‐values were calculated using Student's unpaired *t*‐test (DMSO control vs. ataluren). The white‐colored box graphs represent the normal sample, while the magenta gradient‐colored graphs represent NF‐09, NF‐11, NF‐25, and NF‐31 in that order.

The readthrough efficiency of ataluren varies depending on the genetic sequence context [11]. In our experiments, *NF1^NS/+^* fibroblasts were divided into two groups, ataluren‐responsive and nonresponsive, based on the change in active RAS levels and MEK‐ERK activity in the presence of ataluren. To understand the molecular processes underlying the readthrough by ataluren, the transcriptome profiles were compared between the two groups. Two ataluren‐responsive (NF‐09 and NF‐11) and two nonresponsive (NF‐25 and NF‐31) *NF1^NS/+^* fibroblasts were selected along with a normal control fibroblast (Figure 3A). The responsiveness of ataluren was verified by measuring the ERK activities and neurofibromin expressions in *NF1^NS/+^* fibroblasts after ataluren treatment (Figure 3B). Similarly, the full‐length neurofibromin was restored in three additional *NF1^NS/+^* patient fibroblasts following ataluren treatment (Figure S3). Furthermore, treatment of *NF1^NS/+^* fibroblasts with ataluren resulted in a significant reduction in the viability of ataluren‐responsive cells, but not in nonresponsive cells (Figure 3C). Collectively, in the presence of ataluren, ataluren‐responsive *NF1^NS/+^* fibroblasts restore neurofibromin and lead to a decrease in ERK‐mediated cell proliferation.

A hierarchical cluster analysis and a volcano plot showed distinct transcriptomic profiles in *NF1^NS/+^* fibroblasts compared to normal fibroblasts, based on gene expression data normalized to normal controls (Figures S4 and S5A). A total of 530 genes were significantly upregulated, while 243 genes were downregulated in *NF1^NS/+^* fibroblasts (Figure S5B). Interestingly, the gene clusters with similar expression patterns were shared among *NF1^NS/+^* fibroblasts depending on ataluren responsiveness (Figure S5A). Gene Ontology (GO) functional analysis and Kyoto Encyclopedia of Genes and Genomes (KEGG) pathway enrichment analysis revealed differential expression of genes involved in the PI3K‐AKT, MAPK, RAS, and WNT signaling pathways in *NF1^NS/+^* fibroblasts compared to normal fibroblasts (Figure S5C, D). Following ataluren treatment, the transcriptome‐wide profiles of *NF1^NS/+^* fibroblasts were systematically analyzed and compared with those under DMSO control treatment (Figures S4 and S6). Remarkably, KEGG pathway enrichment analysis revealed a significant downregulation of genes associated with MAPK, Hippo, TGF‐β signaling pathways, cell cycle regulation, and purine metabolism in ataluren‐treated *NF1^NS/+^* fibroblasts (Figure S6D), suggesting that ataluren treatment mitigates the hyperactivated NF1‐related pathways in *NF1^NS/+^* fibroblasts.

Under ataluren treatment, when the gene expression of *NF1^NS/+^* fibroblasts was normalized to that of the normal control, heatmap analysis revealed subsets of genes that were either downregulated or upregulated exclusively in ataluren‐responsive fibroblasts (NF‐09 and NF‐11) compared to nonresponsive fibroblasts (Figure 3D). Among these, 20 genes with known functions were selected, and their mRNA expressions were validated (Figure 3E; Figure S7).

### 2.4 Identification of New Biomarkers in NF1

**FIGURE 4:**
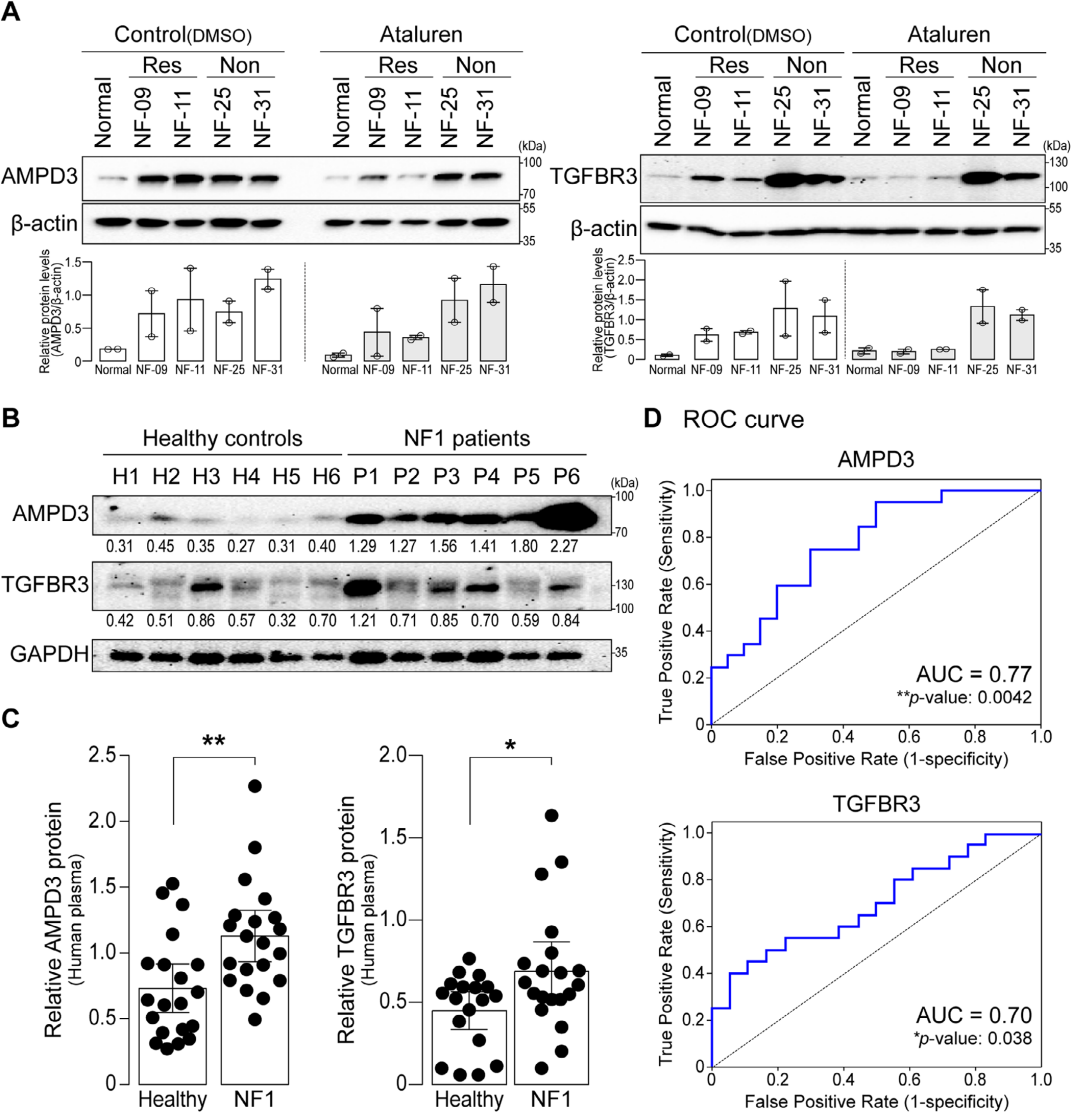
Protein levels of AMPD3 and TGFBR3 are elevated in NF1 patient samples. (A) The protein levels of AMPD3 and TGFBR3 were analyzed in fibroblasts treated with either control or ataluren using Western blotting. Ataluren‐responsive fibroblasts were NF‐09 and NF‐11, and nonresponsive cells were NF‐25 and NF‐31. The level of β‐actin was used as a loading control. Band intensities of the proteins were quantified, normalized to those of β‐actin, and then indicated in the graph below (*n *= 2; mean ± SEM). (B) The protein levels of AMPD3 and TGFBR3 were determined in human plasma samples from NF1 patients and healthy controls. GAPDH serves as an internal control. Band intensities were quantified and calculated as the AMPD3/GAPDH or TGFBR3/GAPDH ratio for each sample presented below. (C) Quantifications of the proteins were normalized to GAPDH and presented as mean ± 95% confidence intervals (*n *= 18–20). Student's *t*‐test with Mann–Whitney test was used (*p < 0.05, **p < 0.01). (D) ROC curve analysis for the new biomarkers AMPD3 and TGFBR3 was conducted based on serum levels in both NF1 patients and healthy controls. AUC: area under the ROC curve.

To identify molecules that are indicative of ataluren responsiveness and functionally associated with neurofibromin and can be detected in blood (plasma) or urine from patients based on their subcellular location, we selected two candidate molecules: adenosine monophosphate deaminase 3 (AMPD3) and transforming growth factor beta receptor 3 (TGFBR3). In line with the transcriptome data (Figure 3D,E), AMPD3 and TGFBR3 were increased in *NF1^NS/+^* fibroblasts compared to normal fibroblasts and then reduced only in ataluren‐responsive *NF1^NS/+^* fibroblasts (Figure 4A). Moreover, the mRNA expression levels of both AMPD3 and TGFBR3 were not significantly altered between the DMSO control and ataluren treatment in normal fibroblasts (Figure S8). Importantly, the abundance of AMPD3 and TGFBR3 was significantly higher in the plasma of NF1 patients compared to healthy volunteers (Figure 4B, C). Furthermore, to evaluate the predictive ability of both AMPD3 and TGFBR3 as potential biomarkers, we conducted a receiver operating characteristic (ROC) curve analysis. The data indicated that both AMPD3 and TGFBR3 exhibited moderate discriminative ability, with area under the curve (AUC) values ranging from 0.70 to 0.77, supporting their potential clinical relevance (Figure 4D). Consistently, we also observed that plasma levels of Ampd3 were significantly elevated in a PN mouse model, *Nf1^flox/flox^;Dhh^cre/+^* (Figure S9). Taken together, these results strongly suggest that AMPD3 and TGFBR3 may serve as novel prognostic biomarkers for monitoring NF1 therapy.

### 2.5 AMPD3 as a Novel Therapeutic Target for NF1

**FIGURE 5:**
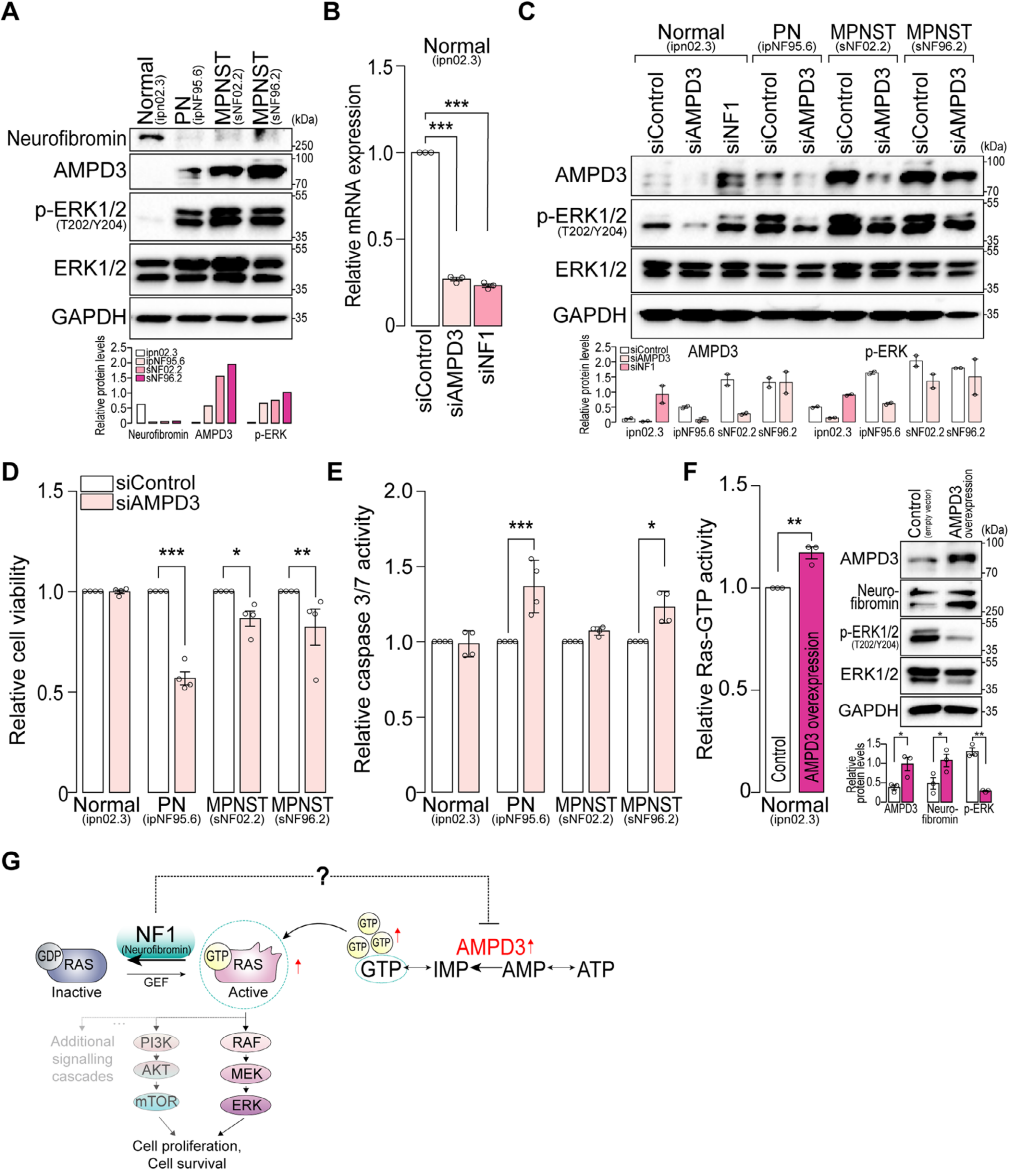
AMPD3 is a potential therapeutic target in NF1‐associated Schwann cell lines. (A) Western blot analysis of the protein levels in 3 NF1‐associated Schwann cell lines, including a normal Schwann cell. Quantification of the proteins was normalized to GAPDH. PN, plexiform neurofibroma cells (ipNF95.6); MPNST, malignant peripheral nerve sheath tumor cells (sNF02.2 and sNF96.2) (B) Relative mRNA expression in siRNA‐transfected Schwann cell lines (ipn02.3) using quantitative PCR (*n *= 3, mean ± SEM; one‐way ANOVA, ***p < 0.001). GAPDH served as an internal control. siControl: control siRNAs, siAMPD3: AMPD3 siRNAs, siNF1: NF1 siRNAs. (C) The protein levels of Schwann cell lines with siRNA transfection were visualized using Western blotting. The protein abundance of AMPD3 and p‐ERK was quantified and normalized to that of GAPDH, which served as a loading control (*n *= 2, mean ± SEM). (D) Cell viability and (E) caspase 3/7‐mediated apoptosis were measured in siRNA‐transfected Schwann cell lines (*n *= 4; mean ± SEM; One‐way ANOVA, *p < 0.05, **p < 0.01, ***p < 0.001). (F: left) The active GTP‐bound RAS (Ras‐GTP) activities of either an empty vector (pcDNA3.1) or the pcDNA3.1‐AMPD3 plasmid‐transfected normal Schwann cells (ipn02.3) were examined using a G‐LISA Ras activation assay. The relative Ras‐GTP activity of AMPD3‐overexpressing ipn02.3 cells was normalized to that of empty vector‐transfected cells (*n *= 3, mean ± SEM). Student's unpaired *t*‐test was used (**p < 0.01). (F: right) Protein levels of AMPD3, neurofibromin, p‐ERK, total ERK, and GAPDH were visualized using Western blotting. Band intensities of the proteins were quantified and presented in the graph below (*n *= 3, mean ± SEM). Student's unpaired *t*‐test was conducted (*p < 0.05, **p < 0.01) (G) Schematic illustration of the correlation between AMPD3 and NF1.

To further investigate whether AMPD3 can be a potential therapeutic target for NF1, its expression was initially examined in four human Schwann cell lines: (1) normal Schwann cells (ipn02.3, *NF1^+/+^*), (2) PN (ipNF95.6, *NF1^NS/−^*), (3) MPNST (sNF02.2, *NF1^+/−^*), and (4) MPNST (sNF96.2, *NF1^−/−^*). The protein level of AMPD3 was markedly higher in NF1‐associated Schwann cells than in normal cells (Figure 5A). Notably, as NF1‐associated malignancy increased in severity, AMPD3 expression and ERK activity were elevated in Schwann cells (Figure 5A). Among the commercially available human Schwann cell lines, only the ipNF95.6 PN cell line contains a germline nonsense mutation (c.2446C>T; p.Arg816Ter) and a somatic nonsense mutation (c.6709C>T; p.Arg2237Ter) in *NF1* [16]. Therefore, we examined the readthrough efficacy of ataluren in *NF1^NS/−^* PN cells (ipNF95.6), and the results exhibited that ataluren did not restore the expression or function of neurofibromin necessary to correct the mutations and improve cell survival in the ipNF95.6 cell line (Figure S10).

Next, we assessed AMPD3 as a promising therapeutic target in NF1. The results showed that siRNA‐mediated AMPD3 repression reduced ERK signaling in NF1‐associated Schwann cell lines (Figure 5B, C). Interestingly, the depletion of neurofibromin enhanced AMPD3 expression and ERK activity in normal Schwann cells (Figure 5C). Moreover, inhibition of AMPD3 significantly decreased cell proliferation and promoted cellular apoptosis in NF1‐associated Schwann cells, but not in normal cells (Figure 5D, E). Conversely, when AMPD3 is overexpressed in normal Schwann cells, the activity of GTP‐bound RAS was significantly enhanced compared with cells expressing an empty vector, which serves as a negative control (Figure 5F). Presumably, as a compensatory mechanism, neurofibromin expression increased in AMPD3‐overexpressing cells, leading to a subsequent decrease in p‐ERK levels (Figure 5F). Collectively, these data demonstrate that AMPD3 expression is inversely correlated with neurofibromin levels (Figure 5G), indicating that AMPD3 could be a promising therapeutic target for NF1.

## 3 Discussion

The current study investigated the therapeutic potential of a PTC suppressor drug, specifically ataluren, in NF1 patients with a nonsense mutation. Our comprehensive and systematic study, using fibroblasts from Korean NF1 patients harboring PTC, demonstrated that ataluren decreased GTP‐bound RAS activity by 22.7% in *NF1^NS/+^* fibroblasts and MEK‐ERK activity in 23.8% of *NF1^NS/+^* fibroblasts. The difference in responsiveness was not related to the location of the NF1 nonsense mutation. The location has not been related to the activity of GTP‐bound RAS [17] either, as we observed that there was no significant difference in the GTP‐bound RAS activities between NF‐09 (p.Arg1748Ter) and NF‐26 (p.Leu993Ter) with and without the GRD domain, respectively. In our study, the difference in responsiveness was more related to the genotype of PTC, UGA, or UAG, but not UAA. These findings indicate that the clinical application of a PTC suppressor should be considered based on the NF1 patient's PTC genotype. In vitro experiments, such as the one performed in our current study, would help predict the clinical efficacy of a PTC suppressor in NF1 patients with PTC.

Nonsense suppression therapy has been proposed for NF1 patients with PTC [18], and ataluren has been selectively and clinically developed to enhance the ribosomal readthrough of PTCs with minimal toxicity [11, 19]. In particular, ataluren selectively rescues target proteins without producing aberrant readthrough products by inducing readthrough of PTCs while preserving normal termination, even at high concentrations [11]. Consistently, we observed that treatment with up to 100 µM of ataluren had no significant effect on cell toxicity in normal fibroblasts (Figure S11). Moreover, five *NF1^NS/+^* patient fibroblasts (NF‐09, NF‐11, NF‐14, NF‐26, and NF‐34) exhibited a substantial restoration of full‐length neurofibromin expression, which consequently reduced cell proliferation following treatment with 100 µM of ataluren.

Recently, two studies investigated the efficacy of PTC suppressors on *NF1* in mouse cortical neuronal cells differentiated from mouse embryonic stem cells with *Nf1^NS/NS^‐3X‐FLAG* [13] and in primary Schwann cells isolated from *NF1^NS/−^* minipig [10]. Using genetically engineered NF1 disease models with a UGA termination codon, PTC suppressors rescued neurofibromin activity, suggesting their potential as therapeutic agents. In our present study, we comprehensively evaluated the efficacy of a PTC suppressor in *NF1^NS/+^* patient fibroblasts with different genotypes and locations of PTC in *NF1*. This allowed us to predict the differential response of a PTC suppressor among various *NF1^NS/+^* patients. As a result, ataluren induced the restoration of full‐length neurofibromin, which acts as a negative regulator of the MEK‐ERK signaling pathway in some, but not all, *NF1^NS/+^* fibroblasts with a UGA or UAG termination codon, supporting the consideration of PTC suppressors for therapeutic purposes in some *NF1^NS/+^* patients. Furthermore, a recent study reported that the therapeutic potential of ataluren was evaluated in a mouse model derived from a patient with NF1 featuring a c.2041C>T; p.Arg681X nonsense mutation. The study revealed that ataluren exhibited age‐ and sex‐dependent efficacy, with lower doses proving effective in females, resulting in a modest reduction in neurofibroma growth and alleviation of paralysis symptoms [14].

In contrast to previous studies that demonstrated the synergistic effect of NMD inhibition in addition to a PTC suppressor by enhancing neurofibromin expression [10, 20, 21], our study did not observe this synergistic effect in several *NF1^NS/+^* patient fibroblasts (Figure S2). Considering that NMD‐susceptible transcripts contain diverse genetic features, such as extended 3′UTRs or 3′UTR exon–exon junctions, and are recognized and degraded by the sophisticated NMD machinery [15, 22], our results indicate that some NF1 transcripts possessing PTCs may not be primary targets of NMD triggers. Moreover, because ataluren competitively prevents the binding of productive release factor‐dependent translational termination, it is expected to have no significant influence on endogenous mRNA levels [11, 23].

Comparing the transcriptomic profiling of *NF1^NS/+^* patient fibroblasts with normal fibroblasts, the MAPK signaling cascades were promoted in *NF1^NS/+^* patient samples. Additionally, genes correlated with NF1 phenotypes, such as skeletal morphogenesis and embryonic organ development, were highly enriched. Furthermore, the physiological phenotypes of NF1‐associated tumors that stimulate fundamental cellular processes were reflected in the *NF1^NS/+^* fibroblasts, such as cell adhesion, proliferation, and migration via RAS, WNT, and PI3K‐AKT signaling pathways [4, 24, 25], and RAS or RAP1‐related signaling pathways, which regulate memory formation in neurons, were also highly overexpressed [26]. However, under ataluren treatment, the downregulated genes were involved in not only NF1‐related pathways, including MAPK and Hippo signaling cascades [24], but also cell proliferation and survival, such as FoxO, TNF signaling, and cell cycle regulation. Notably, the downregulated genes were highly associated with purine metabolism and the TGF‐β signaling pathway in ataluren‐treated *NF1^NS/+^* fibroblasts.

Importantly, the transcriptome‐wide analysis uncovered the distinct genetic features between ataluren‐responsive and nonresponsive *NF1^NS/+^* fibroblasts. In ataluren‐responsive samples, a subset of genes was differentially expressed due to ataluren‐induced restoration of neurofibromin, which was not observed in nonresponsive samples. Among these genes, there are known NF1‐related genes such as platelet‐derived growth factor receptor alpha (PDGFRA) [27, 28], and others that are involved in multiple signaling pathways, including atypical chemokine receptor 4 (ACKR4), DEP domain‐containing mTOR‐interacting protein (DEPTOR), frizzled class receptor 4 (FZD4), mastermind‐like transcriptional coactivator 3 (MAML3), pleckstrin homology and RhoGEF domain‐containing G1 (PLEKHG1), and TGFBR3 [24].

There is a clear consensus that biomarkers play a crucial role in understanding the relationship between biological processes and clinical outcomes [29]. Biomarkers, as surrogate endpoints, can predict the effect of drug treatment. Therefore, we aimed to identify monitorable biomarkers for response to NF1 therapy. Herein, we selected two candidates, AMPD3 and TGFBR3, considering the significant change in their expression in response to the restored expression of neurofibromin and their potential functional connection to neurofibromin. AMPDs (AMPD1, AMPD2, and AMPD3) regulate the nucleotide metabolism of the purine pool by deaminating AMP to inosine monophosphate. Unlike AMPD1 mainly expressed in skeletal muscles, AMPD2 and AMPD3 are ubiquitously expressed; in particular, erythrocytes mostly express AMPD3 [30, 31]. Several reports have demonstrated that the expression levels of AMPDs may serve as prognostic biomarkers in colorectal or head and neck squamous cell carcinoma [32, 33]. Additionally, TGFBR3 (also known as Betaglycan) is involved in cancer progression and has been suggested as a prognostic marker for cancer metastasis [34]. However, the association of AMPD3 and TGFBR3 with NF1 remains unknown. Both AMPD3 and TGFBR3 were detected in human plasma, as confirmed in our study. Compared with normal controls, the protein levels of AMPD3 and TGFBR3 in NF1 patients were significantly elevated and reduced, respectively, in response to restored neurofibromin expression, suggesting that AMPD3 and TGFBR3 could serve as candidate biomarkers for evaluating the effectiveness of a PTC suppressor drug in NF1.

Since AMPD promotes the synthesis of GTP [35], a resource for the active form of RAS (GTP‐bound RAS), AMPD3 can directly or indirectly affect neurofibromin activity. The overexpression of AMPD is highly associated with hyperactive RAS, inhibiting the anticancer activity of neurofibromin that regulates the MEK‐ERK signaling pathway. However, under normal conditions, AMPD3 overexpression leads to a compensatory increase in the activity of wild‐type neurofibromin, resulting in a subsequent decrease in p‐ERK levels. There is additional evidence that AMPD3 is positively correlated with the receptor tyrosine kinase KIT in gastrointestinal stromal tumors, leading to drug resistance [36]. Conversely, the repression of AMPD3 could enhance anticancer activities by restoring neurofibromin expression. Importantly, in our study, the inhibition of AMPD3 reduced the growth of NF1‐associated Schwann cells, whereas it did not affect normal cell survival, suggesting that future research should evaluate AMPD3 inhibitors as potential new therapeutic agents for NF1‐related diseases. Collectively, the interplay between NF1 and AMPD3 is likely to be complex and requires further comprehensive study to be fully elucidated.

Among the commercially available NF1 cell models, the ipNF95.6 PN Schwann cell line is the only one that contains a nonsense mutation (*NF1^NS/−^*). The sNF02.2 MPNST Schwann cell line carries a heterozygous missense mutation (c.4868A>T; p.Asp1623Val) on one allele [37], and the sNF96.2 MPNST Schwann cell line has a frameshift mutation (c.3683delC) on one allele and loss of heterozygosity on the other [37]. In our study, ataluren failed to restore neurofibromin function and did not decrease cell survival in the ipNF95.6 cell line (Figure S10), indicating that the readthrough efficacy of ataluren is influenced by the surrounding genetic sequence context of the PTC [11]. This variability in ataluren response represents a limitation of our study, as it complicates the selection of animal models that adequately reflect the spectrum of therapeutic outcomes. Despite this challenge, we plan to develop *NF1^NS/+^* patient‐derived transgenic mouse models harboring both ataluren‐responsive and nonresponsive mutations identified in vitro, to further evaluate the in vivo efficacy of ataluren.

In the current study, we conducted experimental analyses using primary fibroblasts derived from *NF1^NS/+^* patients. Although fibroblasts do not represent the tumor‐initiating cell population, such as Schwann cells, they offer a genetically relevant model for evaluating cellular responses to NF1 haploinsufficiency and pharmacological interventions in a patient‐specific context. Furthermore, fibroblasts play a critical role in the tumor microenvironment, influencing Schwann cell behavior and tumor progression [38]. Therefore, modulating fibroblast function may yield indirect yet significant therapeutic effects. Importantly, our findings provide proof‐of‐concept for correcting nonsense mutations across diverse PTC genotypes and loci in NF1 patient‐derived fibroblasts.

In conclusion, we comprehensively and systematically evaluated the efficacy of ataluren in fibroblasts of NF1 patients harboring nonsense mutations. Approximately 23% of *NF1^NS/+^* fibroblasts showed significant decreases in GTP‐bound RAS activity and 24% in ERK signaling after ataluren treatment. Based on transcriptome‐wide profiling, we found that both AMPD3 and TGFBR3 expressions were responsive to rescued neurofibromin through ataluren treatment, suggesting that they could serve as potential prognostic biomarkers for monitoring NF1 therapy. Exceptionally, inhibiting AMPD3 is a novel and effective therapeutic strategy for NF1‐related diseases.

## 4 Materials and Methods

### 4.1 Patient‐Derived Fibroblasts and Plasma Samples

Primary fibroblast cells were isolated from skin biopsies of *NF1^NS/+^* patients and healthy controls, as previously described [39]. This study was approved by the Institutional Review Board at the Asan Medical Center (IRB no. 2016‐0768). Human normal Schwann cell lines (ipn02.3), plexiform neurofibroma (PN, ipNF95.6), and 2 MPNST cell lines (sNF02.2, sNF96.2) were obtained from the American Type Culture Collection. The fibroblasts, Schwann cell lines, and HEK293 cells were cultured in DMEM (Gibco) supplemented with 10% (v/v) fetal bovine serum (Gibco) and penicillin‐streptomycin (Gibco).

Human blood samples were obtained from both NF1 patients and healthy volunteers under an approved Human Studies protocol at Asan Medical Center (IRB no. 2019‐0199). Additionally, mouse blood samples were prepared from *Nf1^flox/flox^;Dhh^cre/+^* PN models [40] and normal littermates (*Nf1^flox/flox^*) obtained from Jackson Laboratory. The animal protocol was approved by the Institutional Animal Care and Use Committee of Asan Medical Center (IACUC no. 2023‐30‐075). Plasma was isolated from the collected whole blood and stored in a ‐80°C deep freezer.

### 4.2 Ataluren Treatment

The fibroblasts were seeded at 4 × 10^5^ cells/dish (∼30% confluency) in a 100 mm tissue culture dish (SPL). One day later, the cells were treated with or without ataluren (PTC Therapeutics) and incubated for 72 h. Since ataluren has a relatively short half‐life of 2 to 6 h in human plasma, it was administered daily for 3 consecutive days. At 48 h after the first treatment, the cells were cultured in media containing ataluren under starvation conditions for 24 h. Then, the cells were stimulated with human EGF (Gibco, 100 ng/µL) for 5 min, followed by washing with PBS, and the cells were harvested. The samples were stored at −80°C until they were used for measuring GTP‐bound RAS activity, Western blot analysis, and transcriptome‐wide analysis.

### 4.3 GTP‐Bound RAS Activity

To measure the activities of GTP‐bound RAS, 22 fibroblasts from *NF1^NS/+^* patients were treated with ataluren (0, 70, and 100 µM). After 72 h of incubation, the cells were harvested, and then their GTP‐bound RAS activities, the active form of RAS, were measured by a G‐LISA Ras Activation Assay Biochem Kit (Cytoskeleton) according to the manufacturer's instructions. All samples were performed in triplicate at a minimum.

### 4.4 Western Blot Analysis

The fibroblasts were treated with or without ataluren (100 µM) for a 72‐hour incubation period. The cells were lysed using RIPA buffer (Elpis Biotech) with a protease and phosphatase inhibitor cocktail (Roche). The plasma samples (human and mouse) were diluted 1:10 in DEPC water and mixed with SDS loading buffer (Biosesang). The total protein/plasma samples were separated on 8%–12% (v/v) SDS‐PAGE and then transferred to a nitrocellulose membrane (Amersham). The membrane was incubated with primary antibodies against ERK1/2 (Cell Signaling Technology), phospho‐ERK1/2 (Cell Signaling Technology), Neurofibromin (Novus Biologicals), AMPD3 (Novus Biologicals), TGFBR3 (Cell Signaling Technology), β‐actin (Santa Cruz Biotechnology), and GAPDH (Santa Cruz Biotechnology). Secondary antibodies used were goat anti‐rabbit or anti‐mouse HRP‐conjugated antibodies (Gene Tex). Proteins were detected using picoEPD solution (Elpis Biotech) and analyzed with a ChemiDoc imaging system (Bio‐Rad). The band intensities were quantified using ImageJ software [41].

### 4.5 Transcriptome‐Wide Analysis

Total RNAs were extracted from the fibroblasts that were treated with or without ataluren (100 µM) in two independent experiments using TRIzol reagent (Ambion) according to the manufacturer's protocol. The mRNA sequencing libraries were prepared from at least 5 µg of total RNAs using a TruSeq Stranded mRNA Library Prep Kit (Illumina) and sequenced on a NovaSeq 6000 (Illumina) platform with paired‐end 101 bp reads. The sequencing data were aligned to the human GRCh38 reference using the Bowtie2 aligner. Based on the transcriptome data, the analysis of differentially expressed genes, GO, and KEGG pathways was performed using the DAVID functional tool (https://david.ncifcrf.gov/).

### 4.6 Reverse Transcription‐Quantitative PCR

The isolated RNAs were converted to cDNA using a SuperScript III reverse transcriptase (Invitrogen) with both oligo dT (12–18) primer (Invitrogen) and random hexamer (Invitrogen). The qPCR reaction was conducted using an SYBR Green mixture (Takara) with gene‐specific primers (Table S3) on a QuantStudio 5 real‐time PCR system (Applied Biosystems) following the manufacturer's instructions. Relative mRNA expression of target genes was normalized to that of GAPDH, which served as an internal control. All measurements were conducted in triplicate.

### 4.7 siRNA Transfection

The *NF1^NS/+^* fibroblasts and human Schwann cell lines were seeded on six‐well plates (SPL) and transfected with 50 nM of synthesized two siRNA duplexes (siUPF1, siAMPD3, siNF1) or a siRNA control duplex (Bioneer) using Lipofectamine RNAiMax (Invitrogen). The sequences of synthesized siRNA duplexes are shown in Table S4. After 72 h of incubation, the cells were harvested to estimate mRNA or protein expression.

### 4.8 Cell Viability Assay and Caspase 3/7 Assay

For the cell viability assay of ataluren‐treated *NF1^NS/+^* fibroblasts or ipNF95.6 PN Schwann cell line, the cells were seeded at 2 × 10^3^ cell/well in a 96‐well plate (SPL) and treated with ataluren in a dose‐dependent manner. After 72 h of incubation, cell viabilities were measured using a CCK‐8 assay solution (Dojindo Molecular Technologies) according to the manufacturer's protocols. The assays were carried out in triplicate.

Human Schwann cell lines (normal, PN, and MPNST) were seeded in 96‐well plates (SPL) and transfected with siAMPD3 or siNF1 siRNA duplexes using Lipofectamine RNAiMax (Invitrogen). After 72 h of incubation, cell viabilities were assessed using a CCK‐8 assay solution (Dojindo Molecular Technologies), and caspase 3/7‐mediated apoptotic activities were estimated using a caspase 3/7‐Glo solution (Promega) according to the manufacturer's protocols. All samples were performed in quadruplicate.

### 4.9 Receiver Operating Characteristic Curve Analysis

To evaluate the predictive ability of the biomarkers, ROC curve analysis was conducted using Python with the scikit‐learn library. The ROC curve was generated by plotting the true positive rate (sensitivity) against the false positive rate (1‐specificity) at various threshold levels. The area under the ROC curve (AUC) was computed to determine the ability of the biomarkers to distinguish between NF1 patients and healthy controls. *p*‐values were calculated using statistical tests for the ROC curve by GraphPad Prism 6 software.

### 4.10 AMPD3 Overexpression

Human *AMPD3* coding sequences (NM_000480) were cloned into the pcDNA3.1 vector (Invitrogen). A normal Schwann cell line (ipn02.3) was seeded in a 100 mm tissue culture dish (SPL) at approximately 30% confluency. One day later, the AMPD3 plasmids or empty pcDNA3.1 vectors (control) were transfected into the cells using Lipofectamine 3000 (Invitrogen) according to the manufacturer's protocol. After 72 h of incubation, the cells were harvested to assess GTP‐bound RAS activity and to perform Western blotting.

### 4.11 Statistics

All statistical analyses were conducted using paired or unpaired Student's *t*‐test, and one‐way or two‐way ANOVA by GraphPad Prism 6 software. Statistical significance was set at *p < 0.05, **p < 0.01, and ***p < 0.001.

## Author Contributions

Soyoung Kim, Hyosang Do, and Beom Hee Lee conceived and designed the experiments. Soyoung Kim, Hyosang Do, Sun Hee Heo, and Minji Kang performed the experiments and analyzed the data. Soyoung Kim, Hyosang Do, Soojin Hwang, Dohyung Kim, Min‐Hoo Chang, Kyung Kim, and Beom Hee Lee discussed the results. Beom Hee Lee, Min‐Hoo Chang, and Kyung Kim acquired funding. Beom Hee Lee conceptualized and supervised the project. Soyoung Kim and Beom Hee Lee wrote the manuscript. All authors have read and approved the final manuscript.

## Funding

This study was supported in part by Humanscape, Inc., Seoul, Republic of Korea, and the Bio and Medical Technology Development Program of the National Research Foundation (NRF), funded by the Korean government (grant number: NRF‐2022R1A2C2091689) and the Asan Institute for Life Sciences, Seoul, Republic of Korea (2023IP0129, 2024IF0023).

## Conflicts of Interest

This study was supported by Humanscape Inc. Min‐Hoo Chang and Kyung Kim are employees of Humanscape Inc. and Genoscape Pte. Ltd., but have no additional financial or nonfinancial interests to declare. The funders (Humanscape Inc.) had no role in the design of the study; in the collection, analyses, or interpretation of data; in the writing of the manuscript; or in the decision to publish the results. The remaining authors declare no conflicts of interest.

## Ethics Approval Statement

This study was approved by the Institutional Review Board at Asan Medical Center (IRB No. 2016‐0768, IRB No. 2019‐0199). Informed consent was obtained from all participants in the study. The animal protocol included in this study was approved by the Institutional Animal Care and Use Committee of Asan Medical Center (IACUC No. 2023‐30‐075).

## Supporting information




**Supporting Table S1**: Clinical profiles of Korean patients with Neurofibromatosis type 1 (NF1).
**Supporting Table S2**: Overview of clinical characteristics in Korean patients with NF1.
**Supporting Table S3**: Primers used for quantitative PCR.
**Supporting Table S4**: Synthesized siRNA duplexes.
**Supporting Figure S1**: Vimentin and E‐cadherin mRNA levels in primary fibroblasts and HEK293 cells. Relative mRNA expressions of six representative primary fibroblasts included in the study and epithelial HEK293 cells were analyzed using RT‐qPCR (*n *= 3, mean ± SEM). (A) Vimentin and (B) E‐cadherin mRNA levels were normalized to GAPDH levels, which served as an internal control. The *p*‐value was calculated using one‐way ANOVA (***p < 0.001).
**Supporting Figure S2**: Inhibition of the NMD pathway did not show a significant effect on NF1 expression in both ataluren‐responsive and nonresponsive *NF1^NS/+^* fibroblasts. (A) The mRNA expression of NF1 in six representative *NF1^NS/+^* patient fibroblasts and normal fibroblasts was measured using RT‐qPCR. The relative mRNA levels of NF1 in *NF1^NS/+^* patient fibroblasts were normalized to those in normal controls (*n *= 3, mean ± SEM). The *p*‐value was calculated using one‐way ANOVA (***p < 0.001). NF‐09 (c.5242C>T; p.Arg1748Ter) and NF‐11 (c.2560C>T; p.Gln854Ter) were selected as ataluren‐responsive fibroblasts (Res). NF‐22 (c.1381C>T; p.Arg461Ter), NF‐23 (c.4537C>T; p.Arg1513Ter), NF‐25 (c.6792C>A; p.Tyr2264Ter), and NF‐31 (c.3565C>T; p.Gln1189Ter) were chosen as nonresponsive fibroblasts (Non) with PTCs at various loci. (B) Under UPF1‐depleted conditions (siUPF1 transfection), the mRNA levels of UPF1 and NF1 were normalized to those of GAPDH in *NF1^NS/+^* patient fibroblasts. Then, the relative mRNA expression of NF1 in *NF1^NS/+^* fibroblasts was normalized to that of each siControl‐transfected fibroblast. (C) The protein abundance of neurofibromin and p‐ERK was quantified in DMSO control or ataluren treatment (100 µM) using Western blot analysis, with GAPDH serving as a loading control.
**Supporting Figure S3**: Expression of neurofibromin in *NF1^NS/+^* fibroblasts after ataluren treatment. Protein levels of neurofibromin and GAPDH in DMSO control or ataluren (100 µM)‐treated fibroblasts were visualized using Western blot analysis. The primary antibody that recognizes the C‐terminal of neurofibromin (NOVUS) was used. The numbers in red (NF‐14, NF‐26, and NF‐34) indicate the ataluren‐responsive *NF1^NS/+^* patient fibroblasts. Band intensities were quantified, and the neurofibromin/GAPDH ratio was calculated for each sample presented below.
**Supporting Figure S4**: A schematic overview of transcriptomic data analysis. (1) The analysis of the transcriptomic profiling of fibroblasts from each *NF1^NS/+^* patient normalized to that of normal fibroblasts. (2) Following the analysis (1), transcriptomic analysis of ataluren‐treated *NF1^NS/+^* fibroblasts was conducted with results normalized to DMSO‐treated controls.
**Supporting Figure S5**: Transcriptome‐wide analysis of normal and *NF1^NS/+^* patient fibroblasts before ataluren treatment. (A) The diagram presents the results of a two‐way hierarchical clustering of DEGs in four *NF1^NS/+^* patients and normal fibroblasts. The color scale from yellow to blue represents the values of log_2_ (fold change) from large to small. (B) The volcano plot shows the fold change (*x*‐axis) versus the significance (y‐axis) of DEGs in *NF1^NS/+^* patient samples compared with normal controls. (C) GO functional analysis and (D) KEGG pathway enrichment analysis of 91 up‐ or downregulated genes (at least 2‐fold changes) were conducted using the DAVID tool. Statistical significance was set at *p < 0.05, **p < 0.01, ***p < 0.001.
**Supporting Figure S6**: Transcriptome‐wide analysis of normal and *NF1^NS/+^* patient fibroblasts after ataluren treatment. (A–C) Otherwise, as in Figure S5. Control: DMSO‐treated fibroblasts. (D) KEGG pathway enrichment analysis of 202 up‐ or downregulated genes (at least 1.2‐fold changes) was conducted using the DAVID functional analysis tool. Statistical significance was set at *p < 0.05, **p < 0.01, ***p < 0.001.
**Supporting Figure S7**: Relative mRNA expression in *NF1^NS/+^* fibroblasts treated with DMSO control or ataluren (100 µM).
**Supporting Figure S8**: Transcriptomic data of both AMPD3 and TGFBR3 in ataluren‐treated normal fibroblasts, normalized to DMSO‐treated controls. The values of log_2_(mRNA fold change) were measured using the methods described in Figure S4. The yellow box indicates the relative mRNA levels of AMPD3 or TGFBR3 in ataluren‐treated normal fibroblasts, normalized to those in DMSO‐treated fibroblasts.
**Supporting Figure S9**: Elevation of protein levels of Ampd3 in serum samples from mice with PN (*Nf1^flox/flox^;Dhh^cre/+^*) compared to normal littermates (*Nf1^flox/flox^*). (A) The protein levels of Ampd3 were measured in the serum of 8 mice (4 *Nf1^flox/flox^;Dhh^cre/+^* PN mice; 4 *Nf1^flox/flox^* normal littermates). Ponceau S staining was used as a loading control. (B) Band intensities of Ampd3 proteins were quantified and presented in a graph (*n *= 4, mean ± SEM). Student's *t*‐test with Mann–Whitney test was used (*p < 0.05).
**Supporting Figure S10**: Ataluren does not restore the expression or function of neurofibromin in the *NF1^NS/−^* PN Schwann cell line (ipNF95.6). (A) The protein expressions of neurofibromin, AMPD3, p‐ERK, ERK, and GAPDH were monitored using Western blotting. Normal Schwann cell line (ipn02.3) served as a normal control. (B) Band intensities of the proteins were quantified, normalized to those of GAPDH, and indicated in the graph (*n *= 2, mean ± SEM). (C) The cell viabilities of normal (ipn02.3) and PN (ipNF95.6) Schwann cells after ataluren treatment were measured using a CCK‐8 assay. The graph displays the mean ± SEM values (*n *= 3).
**Supporting Figure S11**: Cell viability of ataluren‐treated normal fibroblasts. Relative cell viability assays of normal fibroblasts were conducted after ataluren treatment using a CCK‐8 assay. Mean ± SEM values are shown (*n *= 3).

## Data Availability

The data that support the findings of this study are available from the corresponding author upon reasonable request.
