# Supplementary material for: Ataluren‐Induced Functional Restoration of Neurofibromin in Fibroblasts From Neurofibromatosis Type 1 Patients With Nonsense Mutations
Source: MedComm (2020). 2025 Nov 16;6(12):e70485. doi: 10.1002/mco2.70485 (PMC12620556; doi:10.1002/mco2.70485)
Supplement: Supplementary file 1 — Supporting Table S1: Clinical profiles of Korean patients with Neurofibromatosis type 1 (NF1). Supporting Table S2: Overview of clinical characteristics in Korean patients with NF1. Supporting Table S3: Primers used for quantitative PCR. Supporting Table S4: Synthesized siRNA duplexes. Supporting Figure S1: Vimentin and E‐cadherin mRNA levels in primary fibroblasts and HEK293 cells. Relative mRNA expressions of six representative primary fibroblasts included in the study and epithelial HEK293 cells were analyzed using RT‐qPCR (n = 3, mean ± SEM). (A) Vimentin and (B) E‐cadherin mRNA levels were normalized to GAPDH levels, which served as an internal control. The p‐value was calculated using one‐way ANOVA (***p < 0.001). Supporting Figure S2: Inhibition of the NMD pathway did not show a significant effect on NF1 expression in both ataluren‐responsive and nonresponsive NF1NS/+ fibroblasts. (A) The mRNA expression of NF1 in six representative NF1NS/+ patient fibroblasts and normal fibroblasts was measured using RT‐qPCR. The relative mRNA levels of NF1 in NF1NS/+ patient fibroblasts were normalized to those in normal controls (n = 3, mean ± SEM). The p‐value was calculated using one‐way ANOVA (***p < 0.001). NF‐09 (c.5242C>T; p.Arg1748Ter) and NF‐11 (c.2560C>T; p.Gln854Ter) were selected as ataluren‐responsive fibroblasts (Res). NF‐22 (c.1381C>T; p.Arg461Ter), NF‐23 (c.4537C>T; p.Arg1513Ter), NF‐25 (c.6792C>A; p.Tyr2264Ter), and NF‐31 (c.3565C>T; p.Gln1189Ter) were chosen as nonresponsive fibroblasts (Non) with PTCs at various loci. (B) Under UPF1‐depleted conditions (siUPF1 transfection), the mRNA levels of UPF1 and NF1 were normalized to those of GAPDH in NF1NS/+ patient fibroblasts. Then, the relative mRNA expression of NF1 in NF1NS/+ fibroblasts was normalized to that of each siControl‐transfected fibroblast. (C) The protein abundance of neurofibromin and p‐ERK was quantified in DMSO control or ataluren treatment (100 µM) using Western blot analysis, with GAPDH serv [file MCO2-6-e70485-s001.pdf]

SUPPORTING INFORMATION

Ataluren-induced functional restoration of neurofibromin in fibroblasts from Neurofibromatosis Type 1 patients with nonsense mutations

Soyoung Kim, Hyosang Do, Sun Hee Heo, Minji Kang, Soojin Hwang, Dohyung Kim, Min-Hoo Chang, Kyung Kim, and Beom Hee Lee

Table S1. Clinical profiles of Korean patients with Neurofibromatosis type 1 (NF1)

	All patients (N = 22)
**Baseline**	
Male:Female (%)	11:11 (50%:50%)
Age at diagnosis (years), mean ± SD (range)	14.8 ± 13.1 (0.3−46.3)
Follow-up duration (years), mean ± SD (range)	7.5 ± 3.9 (0.3−17.6)
Familial:Sporadic (%)	3:19 (13.6%:86.4%)
**Symptoms and sign related NF1, N (%)**	
Six or more café au lait spots	22 (100%)
Freckling in the axillary/inguinal region	22 (100%)
Cutaneous/subcutaneous neurofibromas	17 (77.3%)
Diffuse neurofibroma	4 (18.2%)
Deep localized/plexiform neurofibroma	14 (63.6%)
Malignant peripheral nerve sheath tumor	1 (4.5%)
Optic pathway glioma	2 (9.1%)
Lisch nodules	17 (77.3%)
Brain low grade glioma	3 (13.6%)
Skeletal abnormalities	12 (54.5%)
**Plexiform neurofibromas [PN, (N = 14, %)]**	
Number of locations per person, mean ± SD	2.4 ± 1.3
Age at first recognition of PN (years), mean ± SD (range)	16.2 ± 13.1 (2.0−46.6)
Head/Neck	6 (42.9%)
Thorax/Paravertebral	8 (57.1%)
Upper extremities/Lower extremities	9 (64.3%)
Abdomen/Pelvis	5 (35.7%)

**Interventions related NF1, N (%)**	
Surgery	5 (22.7%)
Chemotherapy	1 (4.5%)
Radiotherapy	1 (4.5%)
Age at first interventions (years), mean ± SD (range)	14.8 ± 10.2 (3.7−33.5)
**NF1-plus (NF1+), N (%)***	15 (68.2%)

*As described in our previous study,[9] NF1-plus (NF1+) is defined as the most severe form among the various phenotypes of NF1, using the following criteria: the presence of widely distributed diffuse cutaneous neurofibromas, learning disabilities, autism, seizures, cardiac abnormalities, hearing impairments, optic pathway gliomas, and severe plexiform neurofibromas—those exceeding 3 cm in diameter that cause disfigurement, pain, bony destruction, or occur in the para-aortic region—as well as brain tumors, nerve root tumors, and malignant peripheral nerve sheath tumors.

Table S2. Overview of clinical characteristics in Korean patients with NF1

No.	Sex	Age at diagnosis (years)	FHx	Current age (years)	Six or more café au lait spots	Freckling in the axillary or inguinal region	Cutaneous/ subcutaneous neurofibromas	Diffuse neurofibroma	Deep localized/ PN	MPNST	OPG	LN	Brain tumor	Skeletal lesion	Age at first intervention Including surgery, chemotherapy, radiotherapy (years)	Age at recognition of first PN (years)	Location of PN	NF1+
NF-08	M	3.5	X	12	O	O	X	X	X	X	X	O	X	Scoliosis	-	-	-	O
NF-09	M	6	X	14	O	O	O	X	O	X	X	X	X	X	-	11.8	Leg, arm, pelvis	O
NF-10	F	11.3	X	19	O	O	O	X	O	X	X	O	X	Kyphosis, lordosis	-	12.4	Thorax, arm, leg, pelvis	O
NF-11	F	23.8	X	32	O	O	O	X	X	X	X	O	X	Bony dysplasia	-	0	0	X
NF-13	M	4.3	X	14	O	O	O	X	X	X	X	X	X	Skull defect	11.9	0	0	O
NF-14	M	6	O	14.2	O	O	X	X	X	X	X	X	X	X	-	0	0	X
NF-19	M	18.1	X	27.1	O	O	O	X	O	X	X	O	O	Scoliosis	-	18.6	Leg, thorax	O
NF-20	M	1	X	11.2	O	O	X	O	X	X	O	O	X	X	-	0	0	O
NF-21	F	0.3	X	8.3	O	O	X	X	O	X	X	X	X	X	3.7	2	Head/neck	O
NF-22	M	7.6	X	14.9	O	O	O	O	O	X	X	O	X	X	-	7.8	Head/neck, thorax, pelvis	O
NF-23	M	7.5	X	17.2	O	O	O	X	O	X	X	O	X	Scoliosis	13.4	10	Arm,leg	O
NF-24	F	33.4	O	40.8	O	O	O	X	O	X	X	O	X	Dural ectasia	33.5	33.5	Abdomen	X
NF-25	F	40	X	56.3	O	O	O	X	X	X	X	O	X	X	-	0	0	X
NF-26	F	3	X	10.5	O	O	X	X	O	X	X	O	X	Dural ectasia, lordosis	6.3	3.3	Head/neck, paravertebral	O
NF-27	F	18.3	X	29.3	O	O	O	O	O	X	O	O	O	Bony dysplasia	23.4	25.1	Head/neck, paravertebral	O
NF-28	F	8.8	X	16.6	O	O	O	X	O	X	X	O	X	Bony dysplasia	11.4	9.5	Arm, leg, pelvis, abdomen, thorax	O

NF-29	M	46.3	X	53.7	O	O	O	X	O	X	X	O	X	Scoliosis	-	46.6	Leg	X
NF-30	M	3.8	X	21.4	O	O	O	O	O	X	X	O	X	Bony dysplasia	6	4	Head/neck, leg	O
NF-31	M	10.8	X	24.2	O	O	O	X	O	O	X	O	O	X	9.5	12.6	Thorax, arm, leg, paravertebral	O
NF-32	F	18.1	O	24.8	O	O	O	X	X	X	X	O	X	X	-	0	0	X
NF-33	F	28.8	X	32.1	O	O	O	X	O	X	X	O	X	X	28.8	30	Head/neck, paravertebral , arm, leg	O
NF-34	F	24	X	40	O	O	O	X	X	X	X	X	X	X	-	0	0	X

No., Number; M, Male; F, Female; FHx, Family history; PN, Plexiform neurofibroma; MPNST, Malignant peripheral nerve sheath tumor; OPG, Optic pathway glioma; LN, Lisch nodules; O, Presence; X, Absence; -, not indicated; NF1+, NF1-plus

Table S3. Primers used for quantitative PCR

Target genes	5'→3'	Description
ACKR4	GCCTTTTTGGGCTGTTAATG<br>CTGATTGGCTGGGGACTTTAG	Upregulated genes in *NF1*$^{NS/+}$-patient fibroblasts
ADAM23	GTCAGTGCCCACCAAATCTTC<br>CGCAGTTTCCCTTCTCAGTG	
AMPD3	CCATTGCCATGTCTCCTCTTAG<br>GGTGCTCAGCTTCCACACTTG	
CCDC136	GGCTTCTGGTAGTGCAGGAG<br>CATGGCTTGGTCACCTTTTTC	
CTSC	GTGGCAGCTGCTACTCATTTG<br>GGGCGTACTTTCCTGCAATAAG	
DEPTOR	AAGCTGATGAGCCCTGAAAAC<br>TTGTTGGACACATGCTGGATG	
EEPD1	AGGCTCGAAGTCTCTGGACAAC<br>GCATCCTTCTTGCTCCAGTC	
FZD4	CCTGGCCAGAGAGTCTGAAC<br>AGGCTCCTTTTCACCCAGATG	
KY	GCCTTCAAACCCACTGACATC<br>CCCTCCAGGTACACAGCATTC	
MAML3	CTCCACGGCCACTAGTTTAC<br>TGCCGGATTCAGGAGATAAC	
MMP10	GGCTCTTTCACTCAGCCAAC<br>TCCCGAAGGAACAGATTTTG	
PDGFRA	GGAAGCTGTCAACCTGCATG<br>CTTCCTTAGCACGGATCAGC	
PLEKHG1	CATGCTTGTGGAGGTGATTC<br>GATTGCTTGCTTGGCCTTAG	
PTX3	TGGTGGGTGGAGAGGAGAAC<br>TTCCTCCCTCAGGAACAATG	
STC1	TCAGCTGAAGTGGTTCGTTG<br>GACGAATGCTTTTCCCTGAG	
TGFBR3	CCAAGATGAATGGCACACAC<br>CCATCTGGCCAACCACTACTG	
TMEM144	ATGAGCAACAATGGAGCAGAC<br>CCAAGGCAACCAACCATATG	
ZNF474	GAGAATTTGGGTCCCAGTCA<br>AGCCTCGTTAGTTGCCTGAAG	
TGM1	ACTACGGCCAGTTTGACCAC<br>CTCGGGAGTAATCACCAGAC	Downregulated genes in *NF1*$^{NS/+}$-patient fibroblasts
JHP2	CTCTGGCTCCTGGAACTTTG<br>CTTGAAGCCATGTGTCCACTC	

NF1	ATCCCCACCACAATACCAAAC<br>CATCAATTCCAGGCAGGTAC	NMD effect test
UPF1	CAGCGGATCGTGTGAAGAAG<br>GTGATCTTCTCCACGTTCGC	
Vimentin	GAGAACTTTGCCGTTGAAGC<br>TCCAGCAGCTTCCTGTAGGTG	Fibroblast marker
E-cadherin	CGACCCAACCCAAGAATCTATC<br>AGGCTGTGCCTTCCTACAGAC	Epithelial marker
GAPDH	GGAGTCAACGGATTTGGTCGT<br>GACAAGCTTCCCGTTCTCAG	

Table S4. Synthesized siRNA duplexes

siRNAs	5'→3'
siUPF1_#1	CCUGUCCUGGCUGGUCAAGAUCC<br>AUCUUGACCAGCCAGGACAGAU
siUPF1_#2	GAUCAACAAGCUGGAGGAGCUGU<br>AGCUCCUCCAGCUUGUUGAUAU
siAMPD3_#1	GUUCAGCCUUCAUGAGAUGUUAA<br>AACAUCUCAUGAAGGCUGAAAU
siAMPD3_#2	CCUGGCGGAGAAGGUGUUUGCUA<br>GCAAACACCUUCUCCGCCAGAU
siNF1_#1	GAAGGUUGCGCAGUUAGCAGUUA<br>ACUGCUAACUGCGCAACCUUAU
siNF1_#2	CUUACAUCAAUUGGGAAGAUAAC<br>UAUCUUCCCAAUUGAUGUAAUU

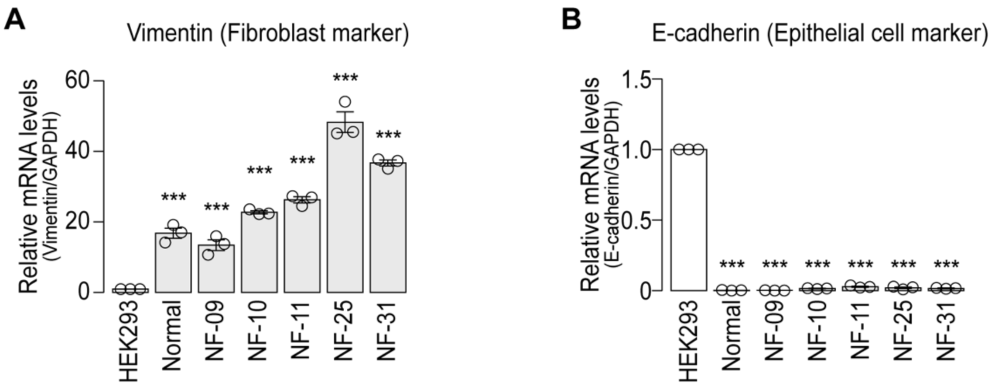

Figure S1. Vimentin and E-cadherin mRNA levels in primary fibroblasts and HEK293 cells

Relative mRNA expressions of 6 representative primary fibroblasts included in the study and epithelial HEK293 cells were analyzed using RT-qPCR (*n*=3, mean ± sem). (A) Vimentin and (B) E-cadherin mRNA levels were normalized to GAPDH levels, which served as an internal control. The *p*-value was calculated using one-way ANOVA (***p<0.001).

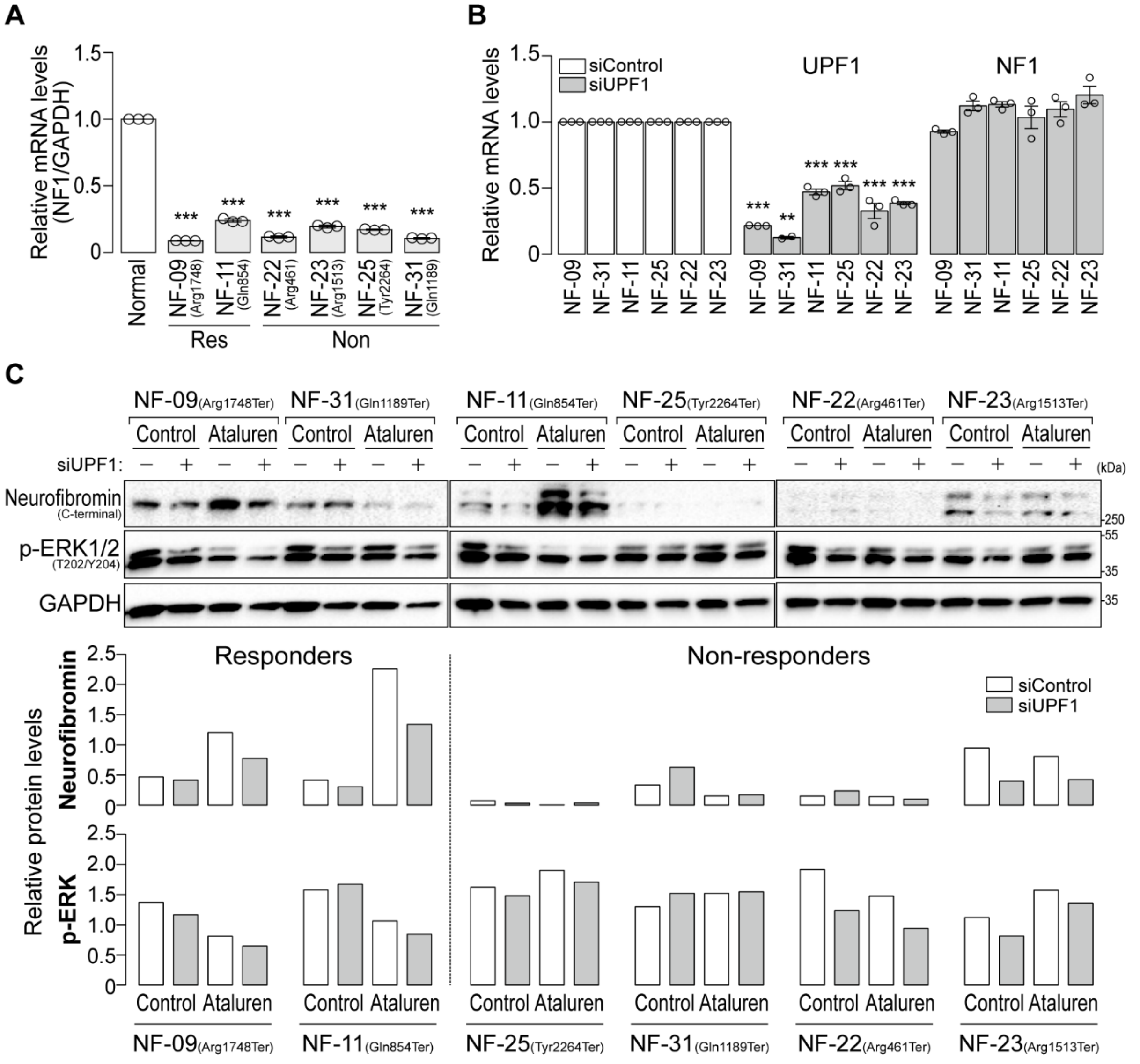

Figure S2. Inhibition of the NMD pathway did not show a significant effect on NF1 expression in both ataluren-responsive and non-responsive *NF1*$^{NS/+}$ fibroblasts

(A) The mRNA expression of NF1 in 6 representative *NF1*$^{NS/+}$ patient fibroblasts and normal fibroblasts was measured using RT-qPCR. The relative mRNA levels of NF1 in *NF1*$^{NS/+}$ patient fibroblasts were normalized to those in normal controls (*n*=3, mean ± sem). The *p*-value was calculated using one-way ANOVA (***p<0.001). NF-09 (c.5242C>T; p.Arg1748Ter) and NF-11 (c.2560C>T; p.Gln854Ter) were selected as ataluren-responsive fibroblasts (Res). NF-22 (c.1381C>T; p.Arg461Ter), NF-23 (c.4537C>T; p.Arg1513Ter), NF-25 (c.6792C>A; p.Tyr2264Ter), and NF-31 (c.3565C>T; p.Gln1189Ter) were chosen as non-responsive fibroblasts (Non) with PTCs at various loci. (B) Under UPF1-depleted conditions

(siUPF1 transfection), the mRNA levels of UPF1 and NF1 were normalized to those of GAPDH in *NF1*$^{NS/+}$ patient fibroblasts. Then, the relative mRNA expression of NF1 in *NF1*$^{NS/+}$ fibroblasts was normalized to that of each siControl-transfected fibroblast. (C) The protein abundance of neurofibromin and p-ERK was quantified in DMSO control or ataluren treatment (100 μM) using Western blot analysis, with GAPDH serving as a loading control.

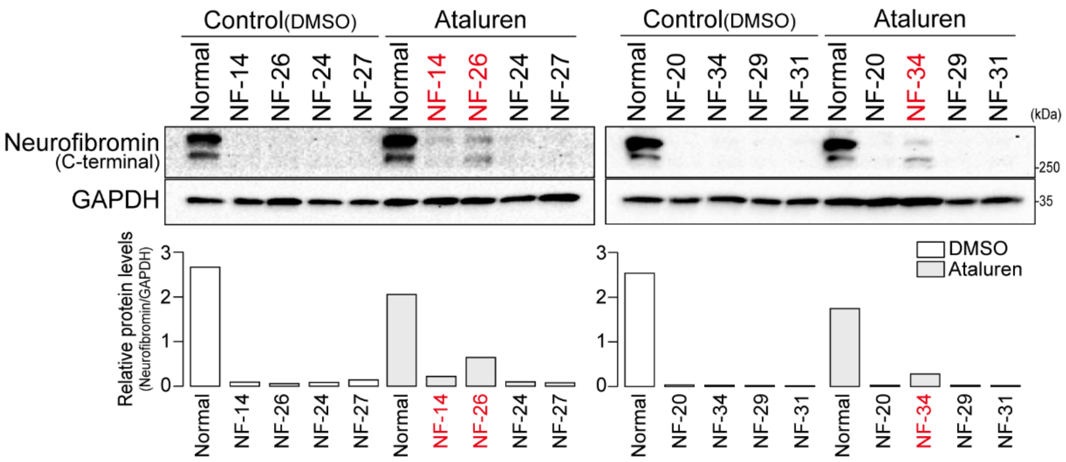

Figure S3. Expression of neurofibromin in *NF1*$^{NS/+}$ fibroblasts after ataluren treatment

Protein levels of neurofibromin and GAPDH in DMSO control or ataluren (100 μM)-treated fibroblasts were visualized using Western blot analysis. The primary antibody that recognizes the C-terminal of neurofibromin (NOVUS) was used. The numbers in red (NF-14, NF-26, and NF-34) indicate the ataluren-responsive *NF1*$^{NS/+}$ patient fibroblasts. Band intensities were quantified and the neurofibromin/GAPDH ratio was calculated for each sample presented below.

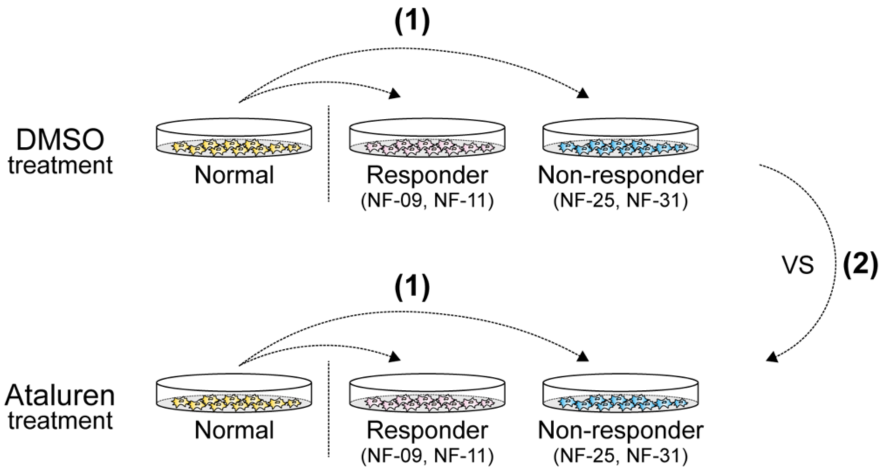

Figure S4. A schematic overview of transcriptomic data analysis

(1) The analysis of the transcriptomic profiling of fibroblasts from each $NF1^{NS/+}$ patient normalized to that of normal fibroblasts.

(2) Following the analysis(1), transcriptomic analysis of ataluren-treated $NF1^{NS/+}$ fibroblasts was conducted with results normalized to DMSO-treated controls.

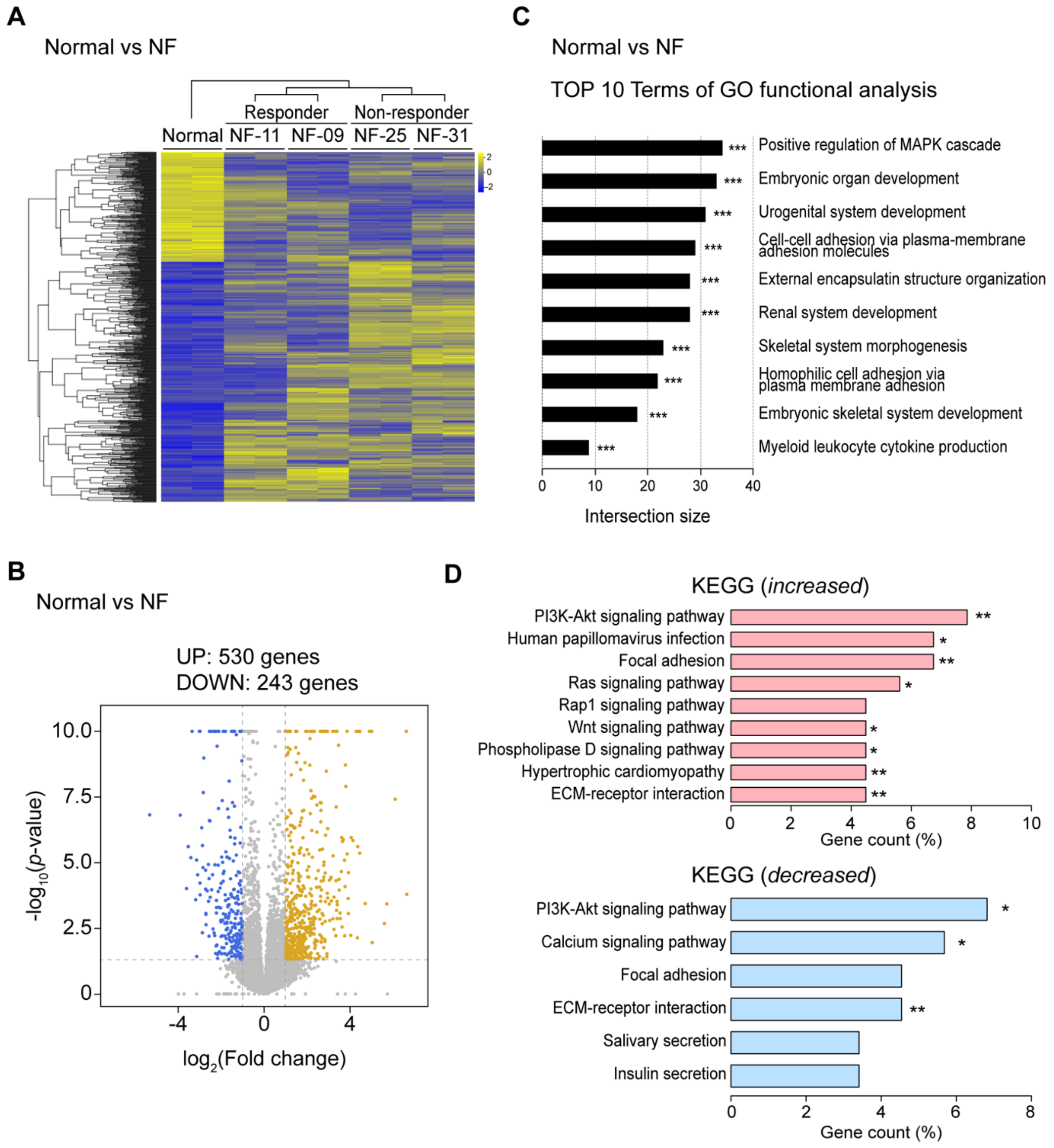

Figure S5. Transcriptome-wide analysis of normal and *NF1*$^{NS/+}$ patient fibroblasts before ataluren treatment

(A) The diagram presents the results of a two-way hierarchical clustering of DEGs in 4 *NF1*$^{NS/+}$ patients and normal fibroblasts. The color scale from yellow to blue represents the values of $\log_2$ (fold change) from large to small. (B) The volcano plot shows the fold change (x-axis) versus the significance (y-axis) of DEGs in *NF1*$^{NS/+}$ patient samples compared with normal controls. (C) GO functional analysis and (D) KEGG pathway enrichment analysis of 91 up or downregulated genes (at least 2-fold changes) were

conducted using the DAVID tool. Statistical significance was set at *$p<0.05$, **$p<0.01$, ***$p<0.001$.

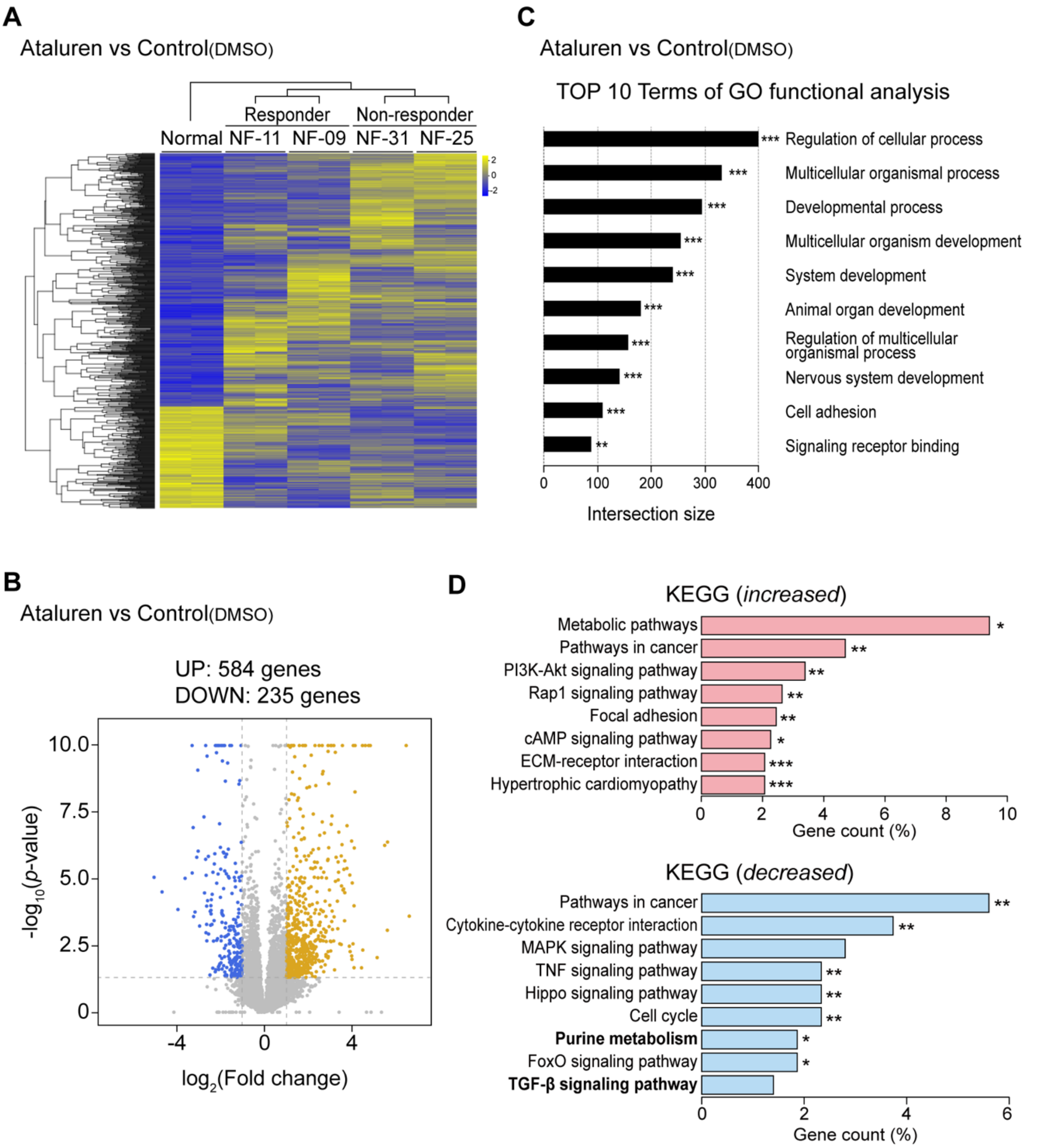

Figure S6. Transcriptome-wide analysis of normal and *NF1*$^{NS/+}$ patient fibroblasts after ataluren treatment

(A-C) Otherwise as in **Figure S5**. Control: DMSO-treated fibroblasts (D) KEGG pathway enrichment analysis of 202 up or downregulated genes (at least 1.2-fold changes) was conducted using the DAVID functional analysis tool. Statistical significance was set at *p<0.05, **p<0.01, ***p<0.001.

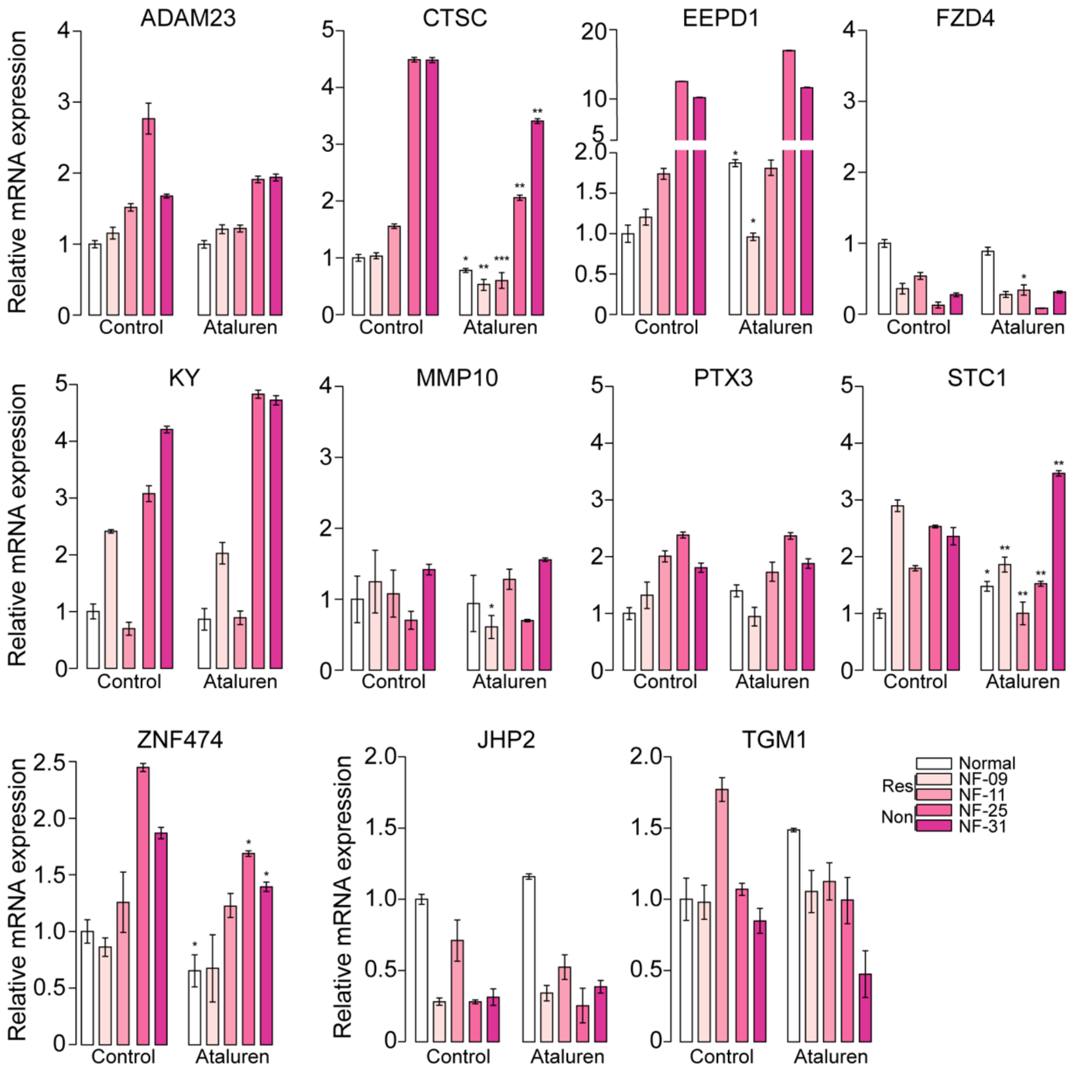

Figure S7. Relative mRNA expression in *NF1*$^{NS/+}$ fibroblasts treated with DMSO control or ataluren (100 µM)

Otherwise as in **Figure 3E**.

Value of log_2(mRNA fold change)

	DMSO control				Ataluren				Normal fibroblast
	NF-09/$_{Normal}$	NF-11/$_{Normal}$	NF-25/$_{Normal}$	NF-31/$_{Normal}$	NF-09/$_{Normal}$	NF-11/$_{Normal}$	NF-25/$_{Normal}$	NF-31/$_{Normal}$	**Ataluren/$_{DMSO}$**
**AMPD3**	2.3553	1.6739	2.1306	3.9593	1.8704	1.3584	2.0795	3.6223	**1.2422**
**TGFBR3**	2.2113	1.5091	3.7519	5.7715	1.1235	1.0681	3.5273	5.8535	**1.0205**

Figure S8. Transcriptomic data of both AMPD3 and TGFBR3 in ataluren-treated normal fibroblasts normalized to DMSO-treated controls

The values of log_2(mRNA fold change) were measured using the methods described in **Figure S4**. The yellow box indicates the relative mRNA levels of AMPD3 or TGFBR3 in ataluren-treated normal fibroblasts, normalized to those in DMSO-treated fibroblasts.

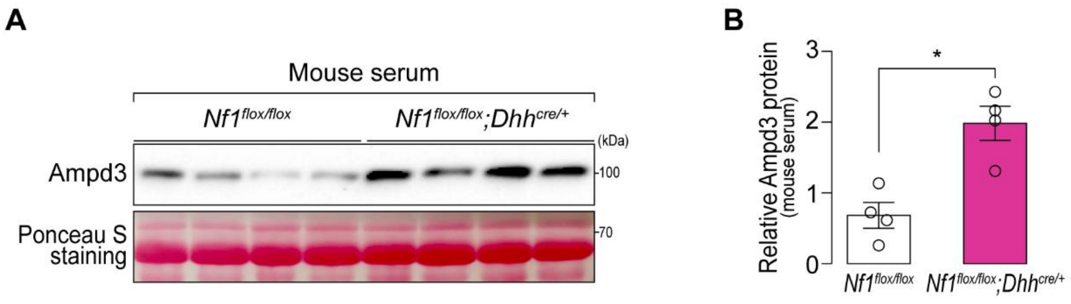

Figure S9. Elevation of protein levels of Ampd3 in serum samples from mice with PN (*Nf1*$^{flox/flox}$*;Dhh*$^{cre/+}$) compared to normal littermates (*Nf1*$^{flox/flox}$)

(A) The protein levels of Ampd3 were measured in the serum of 8 mice (4 *Nf1*$^{flox/flox}$*;Dhh*$^{cre/+}$ PN mice; 4 *Nf1*$^{flox/flox}$ normal littermates). Ponceau S staining was used as a loading control. (B) Band intensities of Ampd3 proteins were quantified and presented in a graph (n=4, mean ± sem). Student's t-test with Mann-Whitney test was used (*p<0.05).

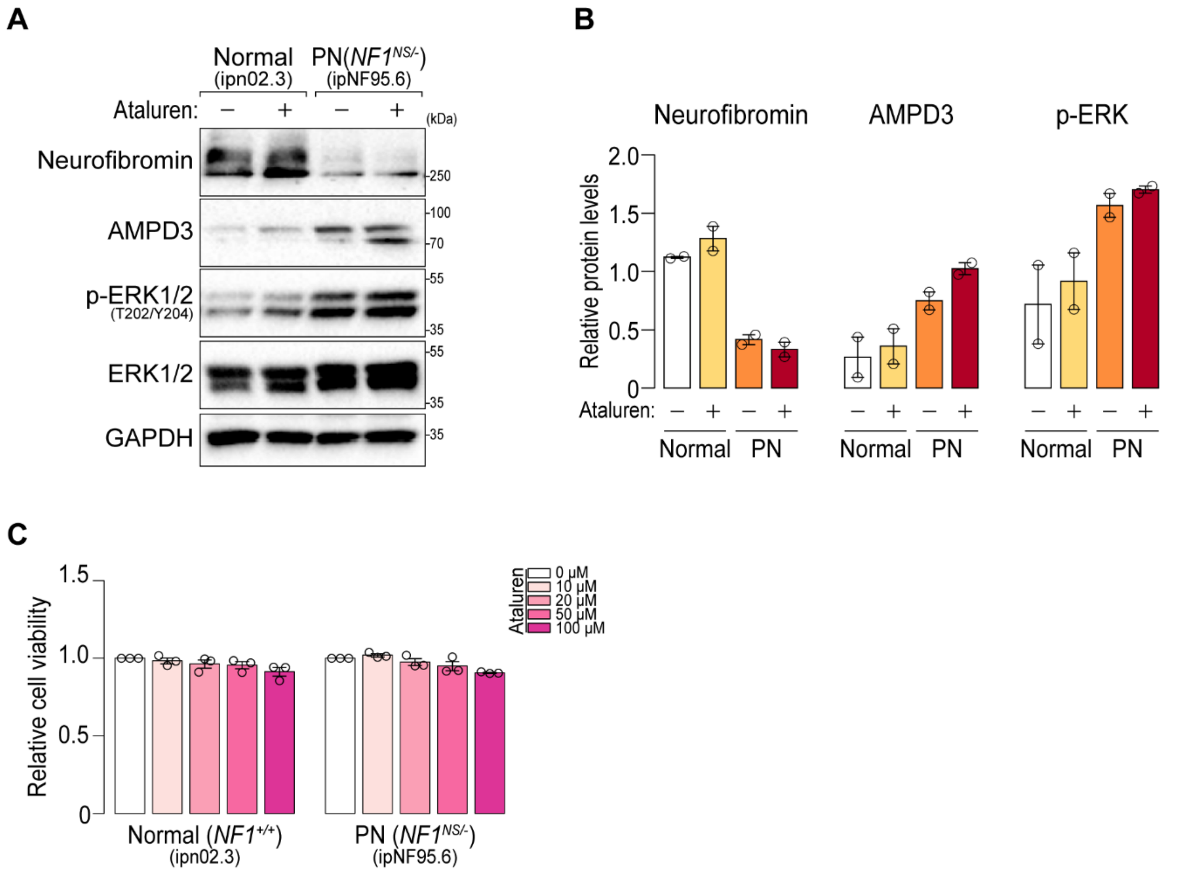

Figure S10. Ataluren does not restore the expression or function of neurofibromin in the *NF1*$^{NS/-}$ PN Schwann cell line (ipNF95.6)

(A) The protein expressions of neurofibromin, AMPD3, p-ERK, ERK, and GAPDH were monitored using Western blotting. Normal Schwann cell line (ipn02.3) served as a normal control. (B) Band intensities of the proteins were quantified, normalized to those of GAPDH, and indicated in the graph (*n*=2, mean ± sem). (C) The cell viabilities of normal (ipn02.3) and PN (ipNF95.6) Schwann cells after ataluren treatment were measured using a CCK-8 assay. The graph displays the mean ± sem values (*n*=3).

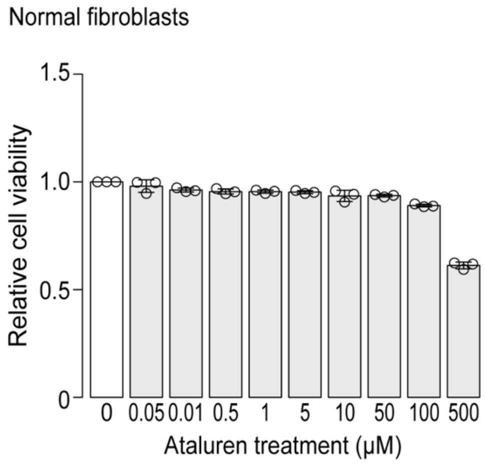

Figure S11. Cell viability of ataluren-treated normal fibroblasts

Relative cell viability assays of normal fibroblasts were conducted after ataluren treatment using a CCK-8 assay. Mean ± sem values are shown (*n*=3).
